# Resynchronized rhythmic oscillations of gut microbiota drive time-restricted feeding induced nonalcoholic steatohepatitis alleviation

**DOI:** 10.1080/19490976.2023.2221450

**Published:** 2023-06-12

**Authors:** Jiafeng Xia, Wanru Guo, Ming Hu, Xiuyuan Jin, Sitong Zhang, Boqiang Liu, Hangyuan Qiu, Kaicen Wang, Aoxiang Zhuge, Shengjie Li, Zhongkang Ji, Lanjuan Li, Kaijin Xu

**Affiliations:** a State Key Laboratory for Diagnosis and Treatment of Infectious Diseases, National Clinical Research Center for Infectious Diseases, Collaborative Innovation Center for Diagnosis and Treatment of Infectious Diseases, the First Affiliated Hospital, School of Medicine, Zhejiang University, Hangzhou, China; b Department of General Surgery, the Second Affiliated Hospital, School of Medicine, Zhejiang University, Hangzhou, China; c Zhejiang Provincial Key Laboratory of Laparoscopic Technology, Sir Run Run Shaw Hospital, School of Medicine, Zhejiang University, Hangzhou, China; d Department of Cardiology, Sir Run Run Shaw Hospital, Zhejiang University School of Medicine, Hangzhou, China

**Keywords:** time-restricted feeding, circadian rhythm, nonalcoholic steatohepatitis, gut microbiota, tryptophan metabolism

## Abstract

With the drive of the endogenous circadian clock and external cues such as feeding behavior, the microbial community generates rhythmic oscillations in composition and function. Microbial oscillations are crucial in orchestrating host metabolic homeostasis during the predictable 24-hour diurnal cycle. A time-restricted feeding (TRF) regimen is a promising dietary strategy to optimize energy utilization, alleviate metabolic syndrome and reinforce microbial cyclical fluctuations. However, the causative relationship between reinforced microbial rhythmicity and TRF-induced metabolic improvement remains elusive. In this study, we corroborated that the TRF regimen notably alleviated obesity and nonalcoholic steatohepatitis (NASH) with reinstated rhythmicity of genera such as *Lactobacillus*, *Mucispirillum*, *Acetatifactor*, and *Lachnoclostridium*. The reshaped microbial oscillations correlate with cyclical fluctuations in intestinal amino acids. Furthermore, fecal microbiota transplantation (FMT) indicated that only the TRF feeding phase-derived microbiota, but not the TRF fasting phase-derived microbiota, could protect mice from NASH and reinstate microbial rhythmicity, confirming that the microbiota improved NASH in a time-of-day-specific manner. The unique role of the TRF-feeding phase-derived microbiota was accompanied by regulation of the serotonergic synapse pathway and rejuvenation of the microbial production of indole derivatives. Our results revealed the discrepant characteristics between the feeding and fasting phases and the time-of-day-specific configuration of microbiota functionality in the TRF regimen.

## Introduction

The mammalian circadian clock is an endogenous system with a regular and precise periodicity, which stably organizes temporal oscillations of biological processes throughout the 24-h period.^1,2^ Subjects with arrhythmic mutations in core oscillators (such as CLOCK, PER, and CRY) are more likely to display metabolic disorders with altered hepatic gluconeogenesis, bile acid, and lipid synthesis.^3–5^ Circadian rhythm disorders dampen diurnal oscillations of metabolic regulators and further lead to the disruption of metabolic homeostasis, which is common in nearly all types of metabolic syndrome (MetS). Adverse behaviors (such as a high-calorie diet, staying up late, overeating, alcohol abuse, etc.) drive the misalignment of central and peripheral rhythms, which presents as phase shifts and amplitude weakening of diurnal oscillation, aggravating the hepatic susceptibility to lipotoxicity and oxidative stress.^6,7^ Diet-induced obesity induces circadian dyssynchrony in the liver, intestinal tract, and adipose tissue, aggravating systemic insulin resistance, hepatic steatosis, and macrophage proinflammatory activation, eventually resulting in metabolic dysfunction such as obesity and nonalcoholic steatohepatitis (NASH).^8^

The gut microbiota maintains a mutualistic relationship with its host and is regarded as the “auxiliary organ” participating in nutrient absorption, immune homeostasis, metabolism, and other physiological activities.^9,10^ Under the coordination of the host circadian clock and feeding behavior, the gut microbiota exhibits cyclical oscillation in composition and function.^11,12^ Influenced by feeding time, diet type, and other environmental conditions, over 60% of the microbial genera periodically oscillate in humans and rodents.^12^ Host clock rhythmicity functionally generates the original entrainment that drives microbial oscillations, as evidenced by the arrhythmicity of microbial fluctuations in clock-deficient mice.^13^ As the most important external cue, the dietary pattern robustly modulates microbial oscillations to synchronize anticipatory food intake behavior. The diurnal oscillations of microbial structure generate the rhythmic fluctuations of microbial metabolites.^12,14,15^ Microbial metabolic products such as short-chain fatty acids (SCFAs) and bile acids (BAs) undergo diurnal oscillations and conversely orchestrate host metabolic homeostasis in time-specific manner.^8,16^ It has been reported that the clock-controlled microbiota mediates the adverse effect of circadian rhythm disorder on nonalcoholic fatty liver disease (NAFLD).^17,18^ Host circadian rhythms are systemically disorganized during the development of high-fat diet (HFD)-induced NASH, which further causes gut dysbiosis and disrupts microbial rhythmicity.^19^ Aberrant microbial oscillations lead to alterations in microbial metabolites, eventually aggravating metabolic dysfunction and hepatic steatosis.

As the peripheral circadian system is particularly sensitive to the feeding pattern, modifications of dietary strategy, for example, intermittent fasting or caloric control, have been regarded as new interventions to reconstitute oscillated rhythms and alleviate MetS. Time-restricted feeding (TRF) is an emerging dietary intervention strategy in which food consumption is restricted to a specific and fixed time window of 8–12 hours on a daily basis.^20,21^ By consistently imposing the diurnal feeding-fasting cycle, the TRF regimen robustly reinforces the rhythmicity of the circadian clock and reprograms metabolic homeostasis and nutrient turnover to couple anticipatory food availability without reducing total calorie intake and physical activity.^3,20,22^ The TRF regimen has beneficial effects in preventing insulin resistance, reducing hepatic lipid accumulation, and maintaining energy homeostasis in the progression of metabolic dysfunction, and these effects even remain in core clock-deficient mice.^3,23,24^ TRF can partially reinstate the rhythmic microbial oscillation in MetS, indicating that TRF may improve metabolic rhythms in a microbiota-dependent manner.^25^

Since the presumptive relationship between TRF, microbial oscillations, and host metabolism is still unclear. We conducted this study to characterize the temporal landscapes of the bacterial communities and their functions, and to further investigate how reinstated microbial rhythms under the TRF regimen contributes to NASH mitigation.

## Results

### TRF improved Western diet-induced obesity and NASH progression

**Figure 1.**
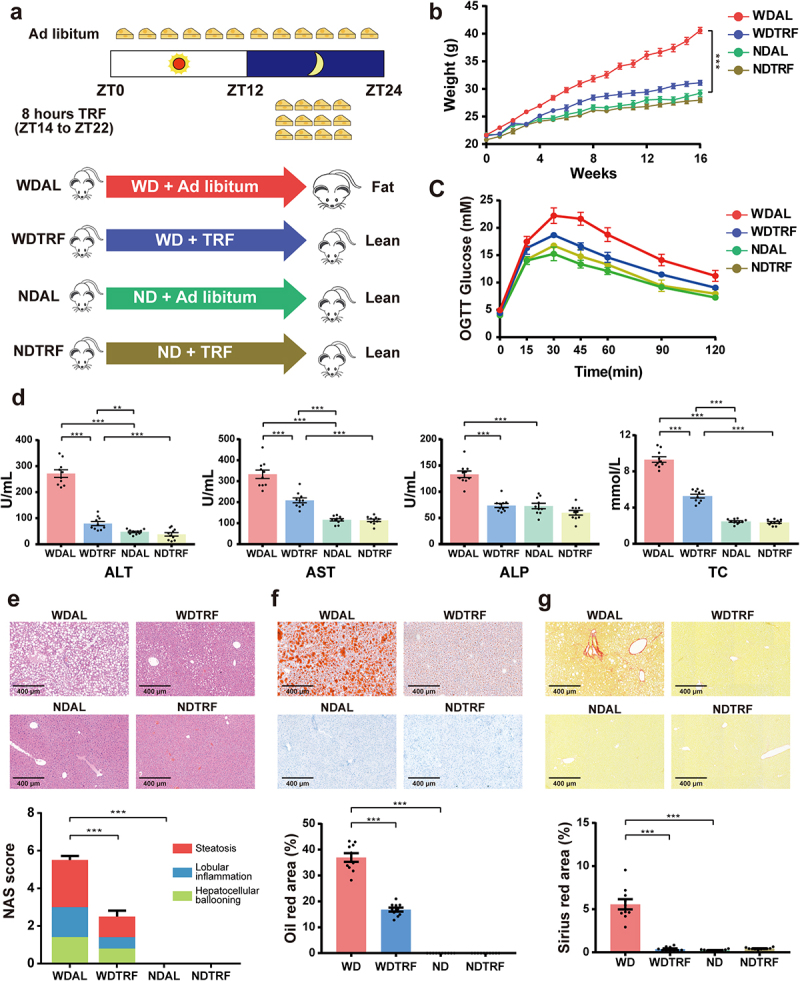
TRF efficiently ameliorated Western diet-induced obesity and NASH progression. (a) Schematic diagram of the animal experimental design. (b) Mouse body weight curve. (c) Curve of blood glucose levels during the OGTT. (d) Serum levels of ALT, AST, ALP and TC. (e) Representative images of H&E staining of liver (upper panel, original magnification × 20, scale bar = 400 mm) and the NAFLD activity score (NAS, lower panel). (f) Representative images of Oil red O staining of liver (upper panel, original magnification × 20, scale bar = 400 mm) and quantitative analysis of the Oil red O area (lower panel). (g) Representative images of Sirius red staining of liver (upper panel, original magnification × 20, scale bar = 400 mm) and quantitative analysis of the Sirius red area (lower panel). *n* = 10 in each group. The data are presented as the mean ± SEM. *p < 0.05; **p < 0.01, ***p < 0.001 for the comparison.

There was no obvious difference in daily food consumption between TRF and ad libitum regimens during the whole 16 weeks of the trial, demonstrating that the 8-hour eating window provided an equivalent amount of energy intake as the ad libitum pattern (Figure S1a). However, mice in the Western diet (WD) TRF regimen (WDTRF) group exhibited a marked reduction in weight gain compared with those in the Western diet ad libitum regimen (WDAL) group (Figure 1b). In addition, the oral glucose tolerance test (OGTT) indicated a noticeable improvement in impaired glucose tolerance in the WDTRF group (Figure 1c; Figure S1b). A similar tendency was observed regarding serum biochemistry, which could reflect the severity of liver injury. As shown in Figure 1d, alanine aminotransferase (ALT), aspartate aminotransferase (AST), alkaline phosphatase (ALP), and total cholesterol (TC) were substantially increased after 16 weeks of WD ad libitum feeding, and TRF significantly decreased those indicators to lower levels. Liver histology revealed signs of extensive hepatocellular ballooning, severe steatosis, and evident inflammation in the WDAL group (Figure 1e). However, only mild steatosis and sporadic inflammatory foci were observed in the WDTRF group. The NAFLD activity score (NAS) was significantly increased in the WDAL group compared with the normal chow diet ad libitum regimen (NDAL) group and partially declined in the WDTRF group. Oil red O staining and Sirius red staining confirmed that the TRF pattern effectively prevented WD-induced excessive lipid droplet accumulation and fibrosis formation (Figure 1f-g). TRF itself did not cause any abnormalities since no differences were observed between the NDAL and normal chow diet TRF regimen (NDTRF) groups. Taken together, the above results suggested that a long-term TRF dietary strategy efficiently ameliorated WD-induced obesity, hyperlipidemia, and NASH progression.

### TRF reshaped the microbiota diurnal oscillation in NASH

**Figure 2.**
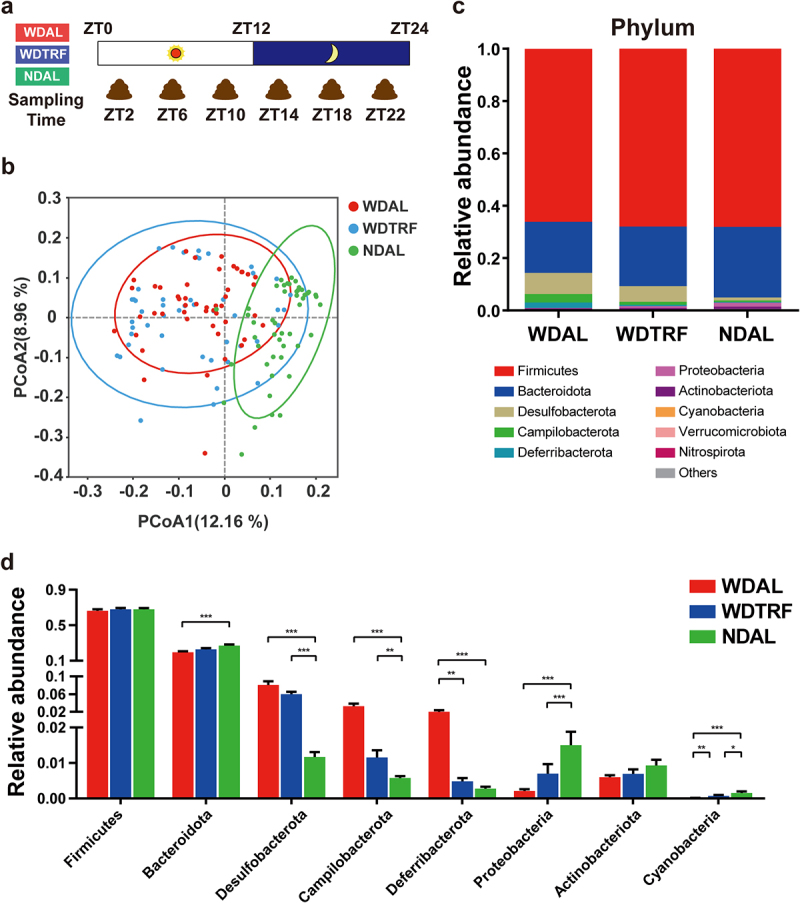
TRF reconstituted the composition of the gut microbiota. (a) Schematic diagram of fecal sampling design over a 24-hour period. (b) Unweighted UniFrac PCoA plot. (c) Stacked bar plot of the relative abundances of phyla with significant changes (Kruskal-Wallis test). (d) Relative abundances of significantly changed phyla (Kruskal-Wallis test). *n* = 48 in each group. The data are presented as the mean ± SEM. *p < 0.05; **p < 0.01, ***p < 0.001 for the comparison.

The gut microbiota is a dynamic mediator between environmental signals and host metabolism. Diurnal fluctuations in microbial structure and function correspond to endogenous rhythmicity in the day-night cycles. To characterize the diurnal rhythmicity of the gut microbiota under different feeding conditions, we collected feces from the WDAL, WDTRF, and NDAL groups at 4-hour intervals over a 24-hour zeitgeber time (ZT) period before sacrifice and conducted fecal 16S rRNA sequencing (Figure 2a).

All stool samples collected in each group across all time points were combined to represent the overall microbial structure of the group. We found that the microbial profiles of the WDAL and WDTRF groups were evidently separated from that of the NDAL group, confirming the WD induced alterations in the microbial community (Figure 2b). At the phylum level, the relative abundances of Bacteroidota, Proteobacteria, and Cyanobacteria were reduced. In contrast, the relative abundances of Desulfobacterota, Campilobacterota, and Deferribacterota were increased in the WDAL group compared with the NDAL group (Figure 2c-d). At the genus level, TRF tended to restore the depleted proportions of *Lactobacillus*, *Muribaculaceae*, *Dubosiella*, *Clostridia_UCG-014*, and *Faecalibacterium* and reduced the excessive proportions of *Faecalibaculum*, *Helicobacter*, and *Mucispirillum* during NASH progression (Figure S2a).

**Figure 3.**
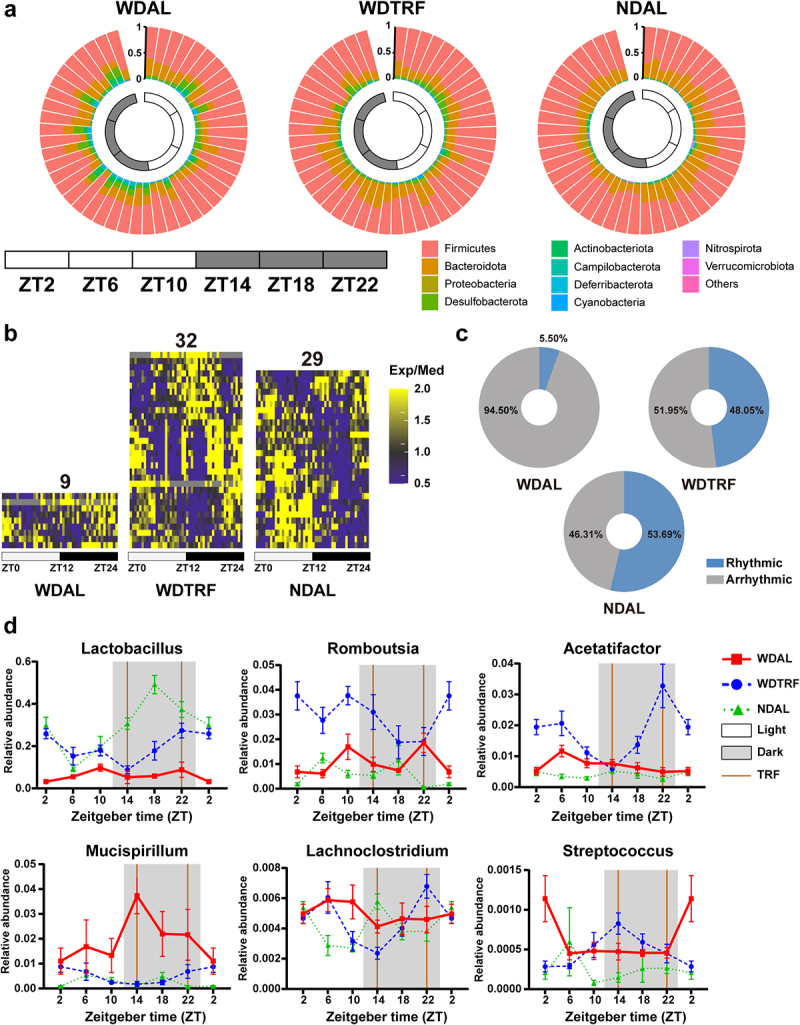
TRF reinstated microbial rhythmicity in WD-fed mice. (a) Dynamic changes in microbial composition at the phylum level over a 24-hour period. (b) Heatmaps of genera with significant rhythmicity in each group. (c) Relative abundance percentages of cyclical and noncyclical genera averaged across all time points. (d) Diurnal oscillations of several genera over the course of a day. *n* = 8 at each time point in each group. The data are presented as the mean ± SEM.

Using the JTK‐cycle algorithm, significantly oscillated taxa were screened according to the criteria *p* < 0.05. At the phylum level, Firmicutes, Bacteroidota, Campilobacterota, and Cyanobacteria featured robust diurnal oscillations in the NDAL group (Figure 3a). No phylum presented diurnal oscillation in the WDAL group, demonstrating the marked disruption of microbial rhythmicity in WD-induced NASH. However, TRF intervention led to profound adjustments in cyclical microbial distribution, as evidenced by the establishment of diurnal oscillations of Bacteroidota and Deferribacterota in the WDTRF group. There were 9, 32, and 29 diurnally oscillated genera in the WDAL, WDTRF, and NDAL groups, respectively. Figure 3b visualizes the phase distributions of these genera across 24 hours. The total proportion of diurnally oscillated genera was substantially reduced by WD feeding (from 53.69% in the NDAL group to 5.50% in the WDAL group). However, it was sufficiently restored by the TRF strategy (from 5.50% in the WDAL group to 48.05% in the WDTRF group), indicating that strict feeding behavior strongly influenced microbial fluctuations (Figure 3c). Notably, the oscillated rhythmicity of potential probiotic genera, such as *Lactobacillus*, *Paraprevotella*, and *Romboutsia*, was abrogated in the WDAL group but restored in the WDTRF group, revealing the predominant effect of rhythmic feeding on these genera (Figure 3d; Figure S2b). Additionally, *Bacteroides* and *Streptococcus* developed de novo oscillations in the WDTRF group. The richness of *Lactobacillus*, *Mucispirillum*, *Acetatifactor*, *Lachnoclostridium*, and *Oscillibacter* continuously decreased and reached a minimum during the fasting period but rebounded and peaked during the feeding period in the WDTRF group. In contrast, *Faecalibaculum*, *Bacteroides*, *Lactococcus*, and *Streptococcus* exhibited the opposite tendency, gradually increasing during the fasting period and dramatically decreasing during the feeding period.

### Rhythmic microbiota orchestrated the dynamic oscillations in intestinal metabolites

Microbiota-produced metabolites maintain dynamic fluctuations corresponding to microbial changes and act as key mediators in microbe-host crosstalk. To determine whether arrhythmic microbiota oscillation perturbed the homeostasis of bacterial metabolites, we next applied an untargeted metabolomics analysis to characterize the landscape of fecal metabolites at all sampling time points.

**Figure 4.**
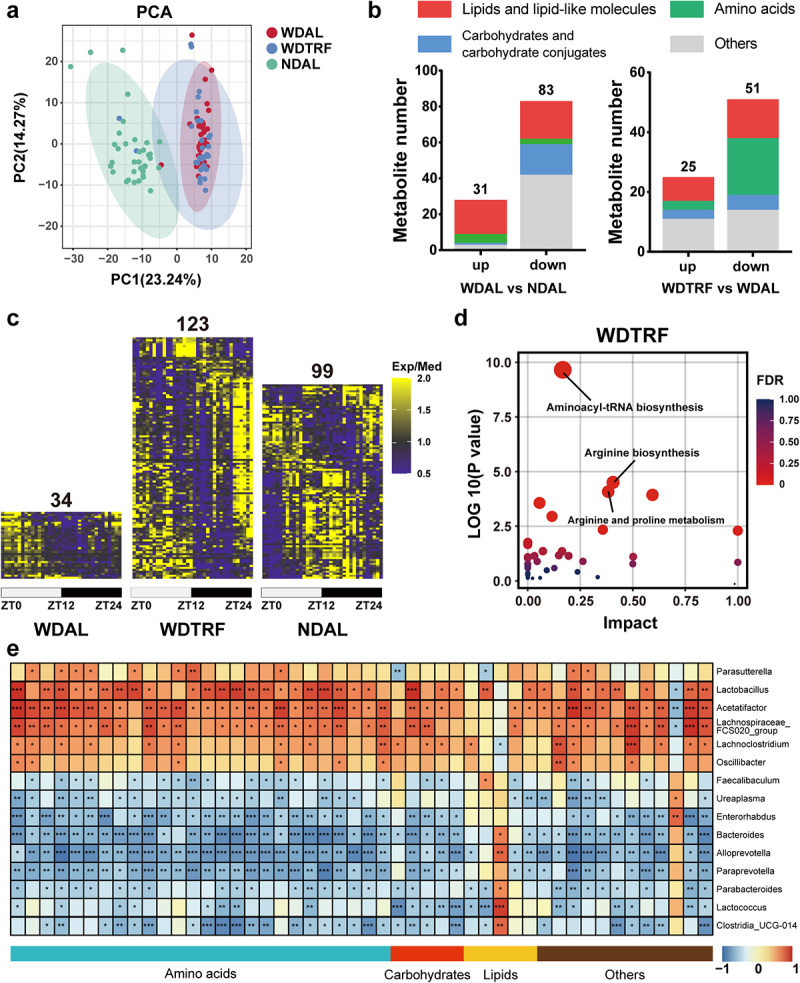
Rhythmic microbiota orchestrated periodic fluctuations in intestinal metabolites. (a) PCA plot of the fecal metabolome. (b) the constitution of differential metabolites in the comparisons of the WDAL vs. NDAL groups and WDTRF vs. WDAL groups. (c) Heatmaps of metabolites with significant rhythmicity in each group. (d) KEGG pathway enrichment analysis of rhythmic metabolites in the WDTRF group. (e) Correlation heatmap of rhythmic genera and rhythmic metabolites. Red represents a positive correlation, and blue represents a negative correlation. *n* = 6 at each time point in each group. *p < 0.05; **p < 0.01, ***p < 0.001 for the comparison.

First, stool samples collected from the WDAL, WDTRF, and NDAL groups were combined to represent the overall metabolite composition of the group. Figure 4a visualizes the clustering of feces collected in all groups, revealing that the metabolome profiles of the WDAL and WDTRF groups were distinctly separated from that of the NDAL group across different time points. We compared the WDAL and NDAL groups, and identified and mapped 31 upregulated metabolites and 83 downregulated metabolites (Figure 4b; Table S3). Lipids and lipid-like molecules were the predominant constituents (*n* = 41), including cholesterol and cholic acid. Carbohydrates were the second richest constituent (*n* = 21), containing galacturonic acid, D-fructose, and D-xylose. Nearly all of them (20/21) were depleted by the Western-style diet. The discrepancy in the metabolite profiles between the WDTRF and WDAL samples was constituted by 76 altered metabolites, mostly belonging to amino acids (*n* = 22) and lipids (*n* = 21). From the above results, we corroborated that food ingredients and feeding regimens strongly influenced nutrient transformation and absorption in the intestine.

Second, we aimed to unravel the cyclical fluctuations in intestinal metabolites under different feeding regimens. Fecal metabolites from each group at different time points during a 24-hour period were used to depict the metabolite rhythmicity of that group. In the NDAL group, 99 metabolites rhythmically oscillated over a 24-hour period (Figure 4c). These metabolites mostly were amino acids involved in aminoacyl-tRNA biosynthesis, glutathione metabolism, and arginine biosynthesis according to the Kyoto Encyclopedia of Genes and Genomes (KEGG) pathway analysis (Figure S3a). Only 34 metabolites showed apparent diurnal rhythms in the WDAL group, suggesting a severe disruption in circadian oscillations of intestinal metabolites.

Notably, 123 metabolites developed robust oscillations in the WDTRF group, demonstrating the reestablishment of circadian rhythm in response to imposed feeding rhythm. Consistent with the NDAL group, rhythmic metabolites in the WDTRF group mainly belonged to amino acids and participated in aminoacyl-tRNA biosynthesis and arginine biosynthesis (Figure 4d).

By matching the microbiome and metabolome results across all time points, we conducted correlation analysis to elucidate the synchronization between rhythmic genera and cyclical metabolites in WDTRF samples. As shown in Figure 4e, circadian alignments of amino acids were positively correlated with rhythmic abundances of *Lactobacillus* and *Acetatifactor* and negatively correlated with those of *Enterorhabdus*, *Bacteroides*, *Alloprevotella*, and *Paraprevotella*. It is worth mentioning that the detected indole derivatives (indole acetic acid, 5-hydroxy-3-indole acetic acid, and tryptamine) were dramatically diminished at all time points in the WDAL group and lost rhythmicity (Figure S3b). However, their rhythmic fluctuations were reshaped with the WDTRF treatment, manifesting as reaching a peak during the feeding period and dampening to the bottom during the fasting period. Altogether, changes in microbial oscillations were accompanied by downstream adjustments in intestinal metabolites.

### The gut microbiota at particular time points was implicated in the TRF-induced NASH mitigation

**Figure 5.**
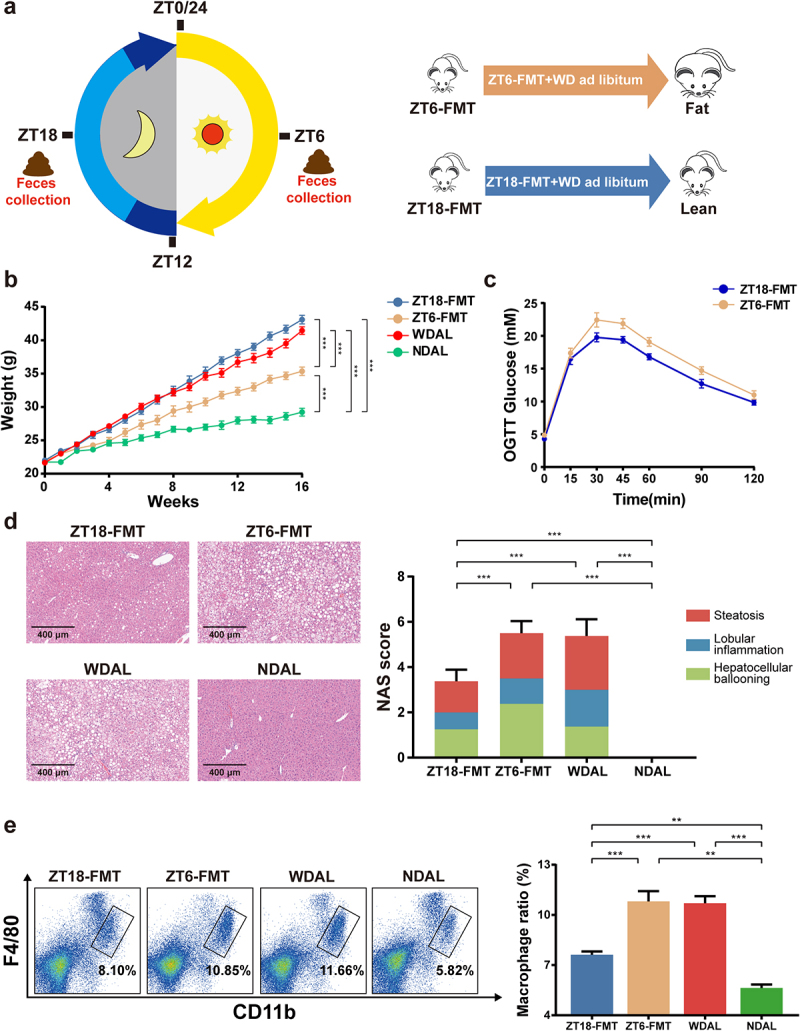
WDTRF feeding phase-derived microbiota alleviated WD-induced steatohepatitis. (a) Schematic diagram of the FMT experimental design. (b) Mice body weight. (c) Curve of blood glucose levels during the OGTT. (d) Representative images of H&E staining of the liver (left panel, original magnification × 20, scale bar = 400 mm) and NAS (right panel). (e) Representative flow cytometry plots of macrophages (left panel) and quantitative analysis of cell percentages (right panel). *n* = 8 in each group. The data are presented as the mean ± SEM. *p < 0.05; **p < 0.01, ***p < 0.001 for the comparison.

The TRF regimen reconstructed periodic oscillations in microbial configurations and metabolites based on feeding behavior, suggesting apparently different microbial functions under saturating or starving conditions. To further determine the beneficial effect of the rhythmic microbiota on MetS and steatohepatitis and to investigate which specific microbial flora contributed predominantly during the daily fluctuation, we next performed fecal microbiota transplantation (FMT). ZT6 (the intermediate time point of the fasting phase) and ZT18 (the intermediate time point of the feeding phase) were considered two representative time points featuring particular microbiota under different conditions. Feces from mice in the WDTRF group were collected at ZT6 and ZT18 and administered to recipients (Figure 5a). All recipients were fed a WD ad libitum for 16 weeks. The WDAL and NDAL groups were recruited as the obesity control and lean control groups, respectively. The lipopolysaccharide (LPS) concentrations in WDTRF ZT6 and ZT18 feces had no obvious difference, confirming that our FMT treatments would not transfer different doses of LPS (Figure S4a). While mice that received ZT6 feces (group ZT6-FMT) showed no improvement in obesity and glucose tolerance, mice that received ZT18 feces (group ZT18-FMT) displayed a noticeable reduction in weight gain and OGTT blood glucose levels compared with these of WDAL or ZT6-FMT (Figure 5b-c; Figure S4b-c). Histological examination indicated that mice receiving the ZT6-FMT treatment developed essentially similar steatohepatitis indicators as those of the WDAL group, revealing the complete ineffectiveness of the WDTRF ZT6-derived microbiota against NASH (Figure 5d). However, compared with mice in the WDAL group, mice in the ZT18-FMT group presented lower NAS scores and reduced Oil red O staining and fibrotic areas (Figure 5d; Figure S5a-b). Consistently, mice transplanted with ZT18 feces showed adequate amelioration of inflammatory macrophage infiltration in the liver, as evidenced by immunohistochemical staining and flow cytometry, proving that the WDTRF ZT18-derived microbiota effectively inhibited inflammatory activation in NASH (Figure 5e; Figure S5c). Despite remaining abnormal compared to the NDAL group, our results still suggested that WDTRF ZT18-derived feces significantly alleviated WD-induced hepatic lipidosis, inflammation, and fibrosis. We also transplanted feces from mice in the group WDAL to recipients, but no NASH-alleviative effect was observed (Figure S4d-e). These results show that specific microbiota during the feeding phase contributed as an independent determinant relative to the effectiveness of TRF in NASH attenuation.

### The gut microbiota from the TRF feeding phase modulated the alterations in hepatic transcriptional expression during NASH

**Figure 6.**
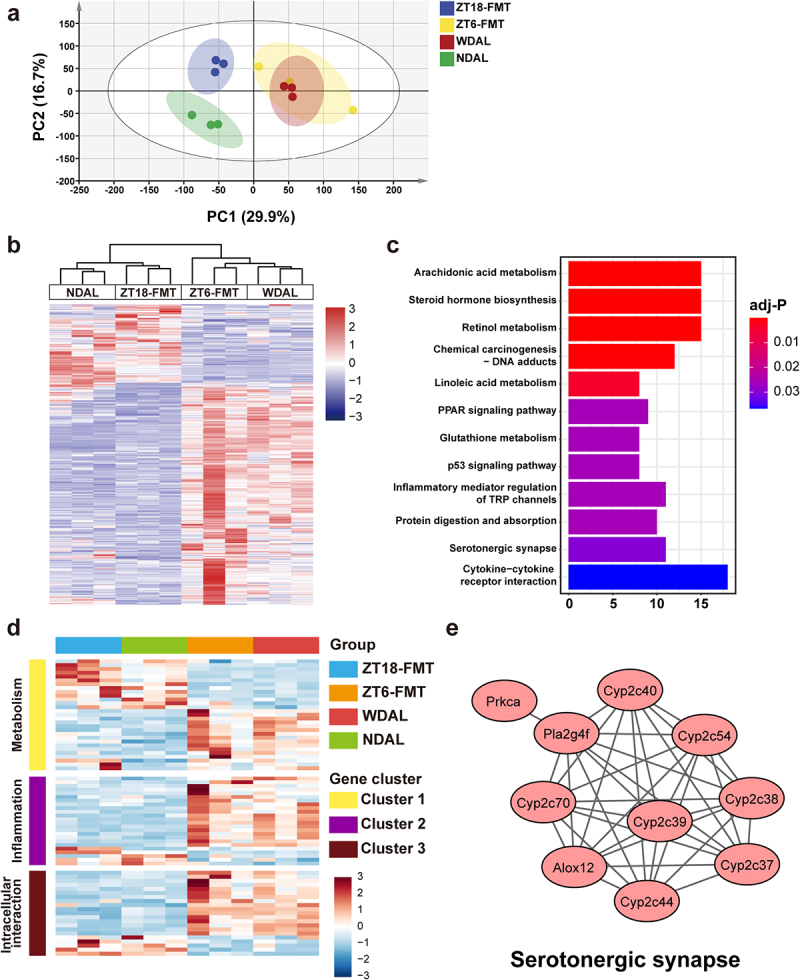
WDTRF feeding phase-derived microbiota modulated the alterations in hepatic transcriptional expression during NASH. (a) PCA plot of the liver transcriptome. (b) Heatmap of DEGs in the ZT18-FMT group versus the ZT-6 FMT. (c) the top 12 pathway terms in KEGG pathway enrichment analysis. (d) Heatmap of the hub genes in three clusters of the PPI network. (e) Visualization of hub genes of serotonergic synapses. *n* = 3 in each group.

As the aforementioned results demonstrate, only the microbiota under TRF feeding conditions, but not the microbiota under TRF starvation conditions, had an alleviating effect on NASH. To further investigate the underlying mechanism, we analyzed liver transcriptome in the ZT18-FMT, ZT6-FMT, WDAL, and NDAL groups. Cluster analysis evaluated transcriptional proximities across samples using principal component analysis (PCA). The transcriptomic profiles of the ZT6-FMT and WDAL groups were highly similar, and their profiles were distinctly distinguished from those of the ZT18-FMT and NDAL groups (Figure 6a). In the comparison between the ZT18-FMT and ZT6-FMT groups, 562 genes were identified as differentially expressed genes (DEGs) based on the criteria *p* < 0.05 and |log2 (fold change)| > 1. A heatmap was used to visualize the hierarchical clustering of these DEGs, further demonstrating the evident discriminations in gene expression patterns among these four groups (Figure 6b). Compared with those in the ZT6-FMT group, 147 and 415 genes were significantly upregulated and downregulated in the ZT18-FMT group, respectively. Pathway enrichment analysis was generated by mapping DEGs to the KEGG database. Twelve pathway terms were screened out using the criterion adj-*p* < 0.05 (Figure 6c). These enriched pathways were mainly assigned to metabolism (such as arachidonic acid metabolism, steroid hormone biosynthesis, and serotonergic synapse), immune response (such as cytokine‒cytokine receptor interaction), and intracellular interaction (such as PPAR signaling pathway and p53 signaling pathway). Seventy-four DEGs were extracted from significant KEGG pathways and subjected to protein-protein interaction (PPI) network analysis. Three interconnected clusters were identified from the PPI network, involving metabolism, inflammation, and intracellular interaction (Figure 6d). Interestingly, hub genes related to serotonergic synapse were enriched in cluster 1, revealing the alteration in physiological function linked to neurotransmission (Figure 6e). Therefore, the gut microbiota of the TRF feeding stage had a unique modifying effect on NASH-related hepatic transcriptomic alterations.

### ZT18-FMT treatment rejuvenated the gut microbiota in NASH mice

**Figure 7.**
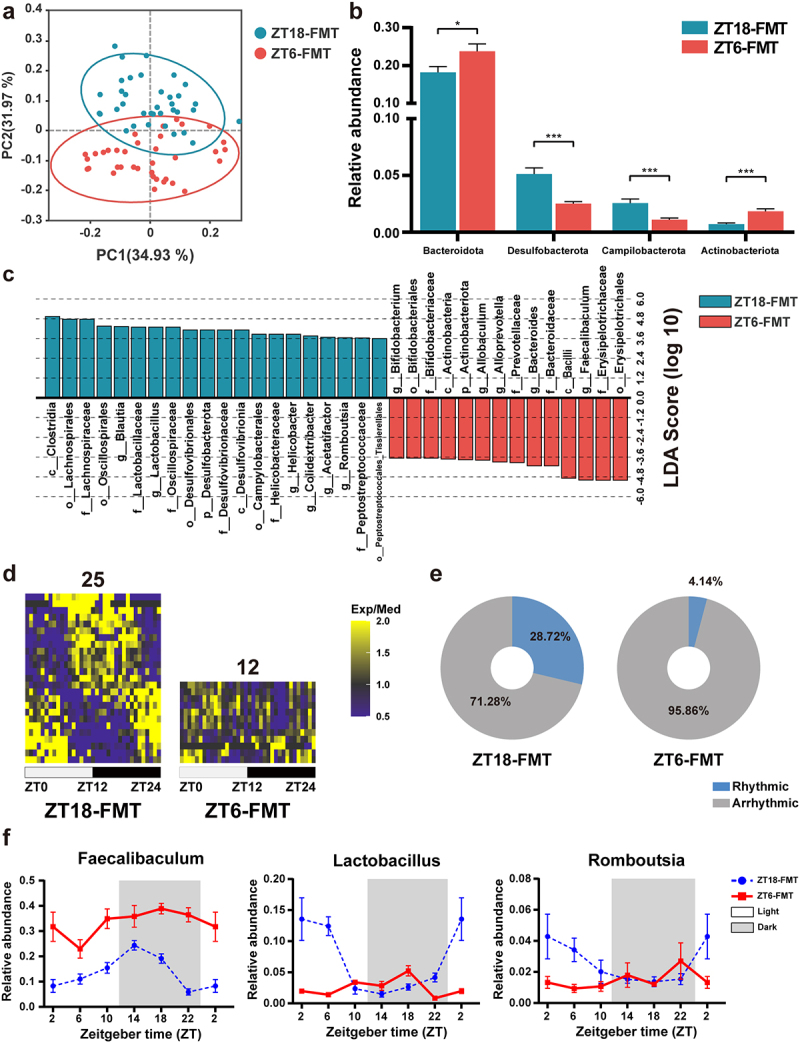
ZT18-FMT treatment modulated the microbial composition and rhythmicity in NASH mice. (a) Unweighted UniFrac PCoA plot. (b) Relative abundances of significantly changed phyla (Mann-Whitney U test). (c) Discriminative taxa with LDA score > 3.5 in LEfSe analysis. (d) Heatmaps of genera with significant rhythmicity in each group. (e) Relative abundance percentages of cyclical and noncyclical genera averaged across all time points. (f) Diurnal oscillations of several genera over the course of a day. *n* = 6 at each time point in each group. The data are presented as the mean ± SEM. *p < 0.05; **p < 0.01, ***p < 0.001 for the comparison.

The microbial variation of recipient mice that received WDTRF ZT6 or ZT18 feces was studied to further clarify the beneficial microbial components contributing to NASH promotion. Principal coordinate analysis (PCoA) intuitively exhibited separate discrimination of the microbial structure between the ZT6-FMT and ZT18-FMT treatments (Figure 7a). The most abundant phyla and genera are provided in Figure S6a-b. At the phylum level, the ZT18-FMT group presented increased relative abundances of Desulfobacterota and Campilobacterota and decreased relative abundances of Bacteroidota and Actinobacteriota compared with the ZT6-FMT group (Figure 7b). At the genus level, the relative abundances of *Lactobacillus*, *Blautia, Dubosiella*, and *Romboutsia* were increased, while the relative abundances of *Faecalibaculum, Bacteroides*, *Allobaculum*, and *Alloprevovotella* were decreased in the ZT18-FMT group compared to the ZT6-FMT group (Figure S6c). Linear discriminant analysis (LDA) effect size (LEfSe) analysis was conducted to identify microbial taxa with significant differences at each hierarchical level from phylum to genus (Figure 7c; Figure S6d). Clostridia, Lachnospiraceae, *Lactobacillus*, *Blautia*, and *Romboutsia* were enriched, whereas Erysipelotrichaceae, *Faecalibaculum*, *Bacteroides*, Prevotellaceae, and *Alloprevotella* were depleted in the ZT18-FMT group compared with the ZT6-FMT group. We next investigated whether FMT treatment changed the microbial oscillations. Interestingly, there were 25 and 12 cyclical genera in the ZT18-FMT and ZT6-FMT groups, respectively (Figure 7d). The total proportion of cyclical genera was significantly increased by ZT18 feces (28.72% in the ZT18-FMT group), while ZT 6 feces transplantation only kept 4.14% cyclical genera (Figure 7e). The rhythmicity of some essential genera, such as *Faecalibaculum*, *Lactobacillus*, *Romboutsia*, *Dubosiella*, and *Lachnospiraceae_UCG-006* was reinstated in the ZT18-FMT group, indicating that feces from a specific time point (intermediate time point of the feeding phase) in the WDTRF group could reshape the microbial structure and rejuvenate the microbial rhythmicity in NASH mice (Figure 7f; Figure S7a).

### The temporal specificity of FMT in NASH mitigation was associated with tryptophan metabolism

**Figure 8.**
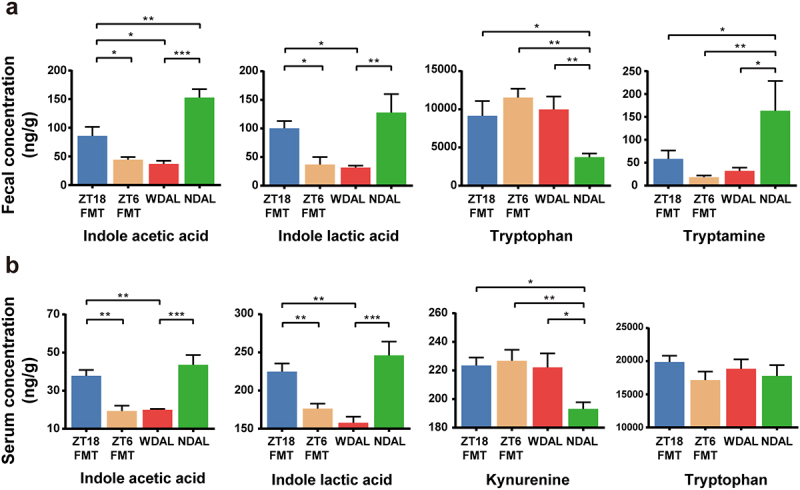
WDTRF feeding phase-derived microbiota rejuvenated microbial production of tryptophan derivatives. (a) Fecal concentrations of tryptophan derivatives (indole acetic acid, indole lactic acid, tryptophan and tryptamine). (b) Serum concentrations of tryptophan derivatives (indole acetic acid, indole lactic acid, kynurenine and tryptophan). *n* = 8 in each group. The data are presented as the mean ± SEM. *p < 0.05; **p < 0.01, ***p < 0.001 for the comparison.

Previous studies have suggested that the biosynthesis and transport of neuroactive metabolites are mediated by intestinal microbiota modulation of tryptophan metabolism. Therefore, we quantitatively detected tryptophan and its derivatives in the serum and feces of ZT18-FMT, ZT6-FMT, WDAL, and NDAL mice. After 16 weeks of WD feeding, the fecal concentration of tryptophan was strikingly elevated. In contrast, the fecal concentrations of indole derivatives (indole acetic acid and indole lactic acid) and tryptamine were diminished (Figure 8a). FMT intervention with WDTRF ZT18-derived microbiota restored the fecal indole acetic acid and indole lactic acid to higher levels. The fecal concentration of indole lactic acid was not significantly different between the ZT18-FMT and NDAL groups, suggesting that its concentration recovered to a near-normal level in the ZT18-FMT group. The ZT-18 FMT, ZT6-FMT, and WDAL groups presented significantly increased serum kynurenine concentrations compared with the NDAL group, indicating the activation of tryptophan-kynurenine conversion (Figure 8b). However, the serum tryptophan concentration and kynurenine to tryptophan ratio were not significantly different among the four groups (Figure 8b; Figure S7b). Comparison between the WDAL and NDAL groups showed that serum levels of indole acetic acid and indole lactic acid were markedly decreased in NASH mice. WDTRF ZT18-derived microbiota effectively promoted the accumulation of these compounds in serum. No difference was observed between the ZT6-FMT and WDAL groups in the above experiments.

## Discussion

Entrainment of internal circadian clocks with external cues (e.g., light, feeding habits, and the sleep-wake cycle) optimizes host adaptation to daily recurring environmental changes, playing a fundamental role in synchronizing physiological and behavioral processes during the predictable 24-hour diurnal cycle.^26^ As an auxiliary organ, the gut microbiota maintains diurnal oscillations in community composition and behavior, which is accomplished with the coordination between host circadian signals and periodic environmental stimuli.^27,28^ A time-restricted feeding (TRF) regimen can reinstate microbial cyclical fluctuations and adapt host metabolism to alternating feeding phases. In this study, we corroborated that the TRF regimen notably alleviated obesity and NASH with reinstated rhythmicity in the composition and function of the gut microbiome. Furthermore, FMT manipulation confirmed the phase-specific role, as only the microbiome during the TRF feeding phase, but not the microbiome during the TRF fasting phase, could prevent the development of NASH.

Feeding behavior is considered the dominant entrainment for peripheral circadian clocks, thereby achieving timekeeping fluctuation of cellular metabolism on a daily basis.^26^ Circadian misalignment, arrhythmic eating patterns, and metabolic disorders often occur together and interact with each other.^5,29^ Erratic lifestyles (e.g., shift work, a high-fat diet, and sleep deprivation) would disrupt circadian integration and increase host susceptibility to metabolic syndrome,^30–32^ suggesting the punctual alignment of feeding behavior with peripheral circadian rhythms participates in the maintenance of metabolic homeostasis. TRF is a strategy of intermittent fasting that controls food intake in 8–10 hours and maintains fasting conditions for at least 14 hours per day without calorie restriction. TRF markedly reshapes nutrient-sensing pathways, energetic systems, and metabolic homeostasis by imposing strict feeding habits.^21,33^ In clinical trials, TRF has exerted its beneficial effects by modulating lipid metabolism, reducing oxidative stress, and optimizing hormone production, consequently delaying aging and prolonging the lifespan.^20,34,35^ In diet-induced obesity (DIO) models, rodents on a liberal obesogenic diet lose cyclical eating habits and consume continuously throughout the 24-hour period,^3^ further dampening rhythmic oscillation of core clock components such as period (Per), cryptochrome (Cry), Clock and Bmal1 in metabolic organs. TRF significantly reduces body weight, improves insulin resistance, ameliorates hyperlipemia, and alleviates the inflammatory response without altering daily activity or food consumption.^17,20,36–38^ Consistent with these studies, our work affirmed that TRF was effective in improving obesity, insulin resistance, lipid deposition, and liver inflammation in 16-week WD-induced NASH. Previous studies have reported that TRF achieves its effectiveness primarily by resetting disrupted circadian rhythms.^21,22,29,39^ Through amplitude augmentation and phase modification of core oscillators, TRF orchestrates transcriptional rhythms of key metabolic regulators, such as pyruvate carboxylase (Pcx), glucose-6-phosphatase (G6pc), and fatty acid synthase (Fasn); reprograms catabolic and anabolic pathways; and promotes nutrient conversion and metabolic homeostasis.^22^ In clock-deficient mice, TRF directly drives periodic oscillations in gene expression and metabolite production, demonstrating that a robust behavioral rhythm can synchronize physiological processes independent of the central clock.^3^

Due to the impact of the gut microbiota on metabolic modulation and immune equilibrium, microbial dysbiosis is recognized as a critical factor in exacerbating lipid accumulation and hepatic inflammation in the pathogenesis of NASH.^9,40–42^ The severity of NASH is linked to gut dysbiosis and shifts in microbial metabolism of nutrients.^41^ The microbiota composition is shifted to an obesity-like status in obesity patients, DIO mice, or ob/ob mice, presenting an increased Firmicutes/Bacteroidota ratio and reduced abundances of potentially beneficial microorganisms.^41,43,44^ We found that probiotic genera such as *Lactobacillus*, *Dubosiella*, *Clostridia_UCG-014*, and *Faecalibacterium* were diminished during NASH progression and restored by the TRF regimen. Supplementation with probiotics can suppress hepatic injury and inflammation in a variety of liver diseases by exerting immunomodulatory properties, restoring the gut barrier, and reprogramming the intestinal metabolome.^45–47^ Although the microbial profile remains stable, it also shows temporal and spatial variability in distribution. As a result of the diurnal rhythmicity of host dietary behavior and metabolic turnover, the gut microbiome also exhibits circadian oscillations. In agreement with a previous study, our results found cyclical oscillations in the NDAL group in Firmicutes and Bacteroidota, two phyla dominating the overall microbial community.^17^ The rhythmicity of most oscillated genera was closely intertwined with circadian variation and feeding behavior, specifically manifesting as accumulation during the dark/active phase and depletion during the light/rest phase or the opposite trend. Mice in the WDAL group displayed completely disrupted microbiota rhythms and random fluctuations. This was attributed to the combined impacts of WD-induced microbial dysbiosis, arrhythmic intake pattern, and disrupted host clock.^48^ Among these reasons, scheduled food consumption is determinant in driving microbial rhythmicity. Food availability can independently entrain microbial rhythmicity in clock mutant mice, and modulation of feeding time leads to the phase shift in the rhythmic bacteria.^12,27^ We found that TRF efficaciously reinstated microbial rhythmicity in WD-fed mice, emphasizing that feeding rhythms could individually stimulate microbiota oscillations. Protective genera such as *Lactobacillus*, *Oscillibacter*, *Romboutsia*, and *Lachnoclostridium* peaked in the feeding phase, whereas obesogenic genera such as *Faecalibaculum*, *Lactococcus*, and Strept*ococcus* peaked in the starving phase. This was an important indication that TRF specifically separated microbial variations on a daily basis according to nutrient availability.

Microbiota diurnal oscillations are crucial in maintaining host physiology and metabolism. Microbiota-derived SCFAs exhibit dynamic fluctuations in response to diurnal oscillations of SCFA-producing bacteria and further activate downstream G protein‐coupled receptors (GPRs) 41/43 in a circadian manner.^49–51^ The oscillated microbiota induces cyclical expression of histone deacetylase 3 (HDAC3) in the intestinal epithelium, which functionally synchronizes the diurnal rhythms of histone acetylation and nutrient uptake in epithelial cells.^52^ Diurnal attachment of segmented filamentous bacteria (SFB) to the intestinal surface generates fluctuations in the expression of antimicrobial proteins, thereby affecting host immune function.^11^ To investigate how microbial rhythmicity influences intestinal homeostasis, we characterized the dynamics of fecal metabolites under different feeding conditions. WDAL and NDAL treatments showed strikingly different nutrient utilization patterns. Although the WD had higher sugar and fat, the WDAL group still showed more absorption of D-fructose, D-xylose, and linoleic acid than that of the NDAL group, which also meant excessive calorie intake and a higher predisposition to adiposity.^17,40,53^ The WDTRF group preferred to excrete derivatives of D-fructose, D-xylose, and cholesterol rather than absorb them. Luminal bioconversion of amino acids was reinvigorated in the WDTRF group, highlighting that a long-term TRF regimen prompted the microflora to optimize energy utilization and chemical biosynthesis. In addition, our results revealed that the fluctuations in specific fecal metabolites presented high concordance with microbial oscillations. Inherent metabolite rhythmicity was misaligned in the WDAL group and reestablished in the WDTRF group. The cyclical metabolites in the NDAL or WDTRF group were largely amino acids and participated in fundamental functions such as aminoacyl-tRNA biosynthesis and arginine biosynthesis. It has been reported that amino acid metabolism is closely entrained to the circadian cycle and that TRF promotes the periodic transcription of amino acid transporters.^54–56^ More importantly, we found that TRF-induced rhythmic genera such as *Lactobacillus*, *Acetatifactor*, and *Enterorhabdus* were positively or negatively correlated with cyclical metabolites, emphasizing the synchronization of microbial bioconversion and amino acid assimilation with periodic microbiota oscillations.

Our study also highlighted the time-of-day-specific configuration of microbiota functionality. Microbial functions associated with nutrient metabolism and DNA repair are enriched in the dark phase, whereas functions associated with detoxification and bacterial chemotaxis are preferred in the light phase.^12,57,58^ TRF-induced circadian variations are triggered by imposing alternative feeding and fasting cycles on organisms. Therefore, TRF-induced rhythmicity features discrepant characteristics between the feeding and fasting phases.^59–62^ Feeding (ZT18) or fasting (ZT6) phase-derived microbiota from the WDTRF group was administered to WD-fed recipients. Surprisingly, only microbiota derived from the feeding condition transferred the NASH-protective phenotype, ascertaining that the microbiota of the WDTRF group improved NASH in a time-of-day-specific manner. The feeding phase-derived microbiota induced higher relative abundances of *Lactobacillus*, *Romboutsia*, and *Dubosiella* and lower relative abundances of *Faecalibaculum* and *Bacteroides* compared with those of the fasting phase-derived microbiota. Their variations were in accordance with changing tendencies in daily compositional oscillations in the WDTRF group. Besides, feeding phase-derived microbiota partially reinstated diurnal oscillations of microbiota, suggesting the strong relationship between microbial rhythmicity and normal physiology.

Drawing on the metabolomics results, we confirmed that fecal aromatic amino acids varied considerably under different feeding conditions due to alterations in tryptophan-metabolizing commensals such as *Lactobacillus*, *Romboutsia*, and *Lachnospiraceae_UCG-006*. Additionally, the unique role of the feeding phase-derived microbiota in improving NASH was highly associated with serotonergic synapses, as evidenced by the transcriptome. Tryptophan can be transformed into kynurenine, serotonin, and indole derivatives with the cooperation of the host and the gut microbiota.^63,64^ Serum kynurenine has been identified as a new diagnostic biomarker for MetS.^65–67^ Indole and its derivatives are only produced by microbial catalysis and are known for their beneficial effects in suppressing inflammation, enhancing the gut barrier function, and ameliorating oxidative stress.^67^ Several indole derivatives can attenuate hepatic lipogenesis and inflammatory infiltration in the pathogenesis of NAFLD.^68,69^ Researchers have noted that intermittent fasting can increase serum concentrations of indole derivatives.^70^ Our study found that transplantation with the feeding phase-derived microbiota led to the accumulation of indole derivatives in both the serum and feces of recipients. At the same time, no such changes were observed for the fasting phase-derived microbiota. Hence, rejuvenated tryptophan metabolism might be the key mediator driving the phase-specific effect of the TRF regimen.

This study has several limitations. First, the gut microbiome has spatial variability from the jejunum to the colon. The microbial rhythmicity in other intestinal segments needs to be explored. Second, FMT treatment using feces derived from other sampling time points (like ZT3, ZT9, ZT15, and ZT21) could better elaborate the dynamic changes in microbial functions.

In summary, our work characterized the composition and function of TRF-induced microbiota rhythmicity and further revealed that only the feeding phase-derived microbiota, not the fasting phase-derived microbiota, presented a NASH-protective phenotype. The NASH-protective effect of the feeding phase-derived microbiota was associated with tryptophan metabolism.

## Materials and methods

### Animals

Six-week-old male specific pathogen-free (SPF) C57BL/6 mice were purchased from Jiangsu Jicui Yaokang Biotechnology Co., Ltd. All mice were maintained in a strict 12-hour light:12-hour dark cycle (lights on at 8:00 a.m.) under controlled temperature and humidity conditions. Before the experiment, all mice were acclimated to a normal chow diet and water ad libitum for 2 weeks. All animal procedures were approved by the Animal Care and Use Committee of the First Affiliated Hospital, School of Medicine, Zhejiang University.

### Experimental model and feeding schedule

Mice were randomly allocated to the following groups: Western diet ad libitum regimen group (group WDAL); Western diet time-restricted feeding (TRF) regimen group (group WDTRF); normal chow diet ad libitum regimen group (group NDAL); and normal chow diet TRF regimen group (group NDTRF). Mice in WDAL and WDTRF groups were fed with western diet (fat 40% kcal, protein 17% kcal, carbohydrate 43% kcal, Research Diets #D12079B, New Brunswick, NJ, USA) and high-sugar drinking water (23.1 g/L d-fructose and 18.9 g/L d-glucose). Zeitgeber time (ZT) 0 was designated lights on, while ZT12 corresponded to lights off. Mice that received the TRF pattern had access to food during the 8-hour window from ZT14 to ZT22. Mice in the WDTRF group were only given water without sugar during the fasting from ZT22 to ZT14. Food intake was monitored daily, and each group’s body weight was measured weekly throughout the experiments. After 16 weeks of the feeding regimen, all mice were sacrificed between ZT0 and ZT4. A schematic illustration of the animal experimental design is shown in Figure 1a.

### Fecal microbiota transplantation

Before FMT treatment, all recipient mice were intragastrically administered an antibiotic cocktail (200 mg/kg ampicillin, 200 mg/kg metronidazole, 200 mg/kg neomycin sulfate, and 100 mg/kg vancomycin) for 7 consecutive days to deplete indigenous intestinal microbiota. During the 16-week experimental period, 350–400 mg of fecal pellets were collected daily from donor mice in the WDTRF group at ZT6 and ZT18. After collection, fresh feces were resuspended in 2 mL of sterile normal saline (NS) and vigorously homogenized, followed by centrifugation (400 × g, 10 min) to precipitate large particles. The supernatant was immediately used for further transplantation. Each recipient was treated with 200 µL of fecal supernatant by gavage daily while receiving WD feeding for 16 weeks.

### Oral glucose tolerance test

OGTT was performed 14 weeks after the feeding regimen. Following 16 hours of fasting (from ZT22 to ZT14), mice were administered 2 g/kg glucose intraperitoneally. Blood samples were obtained from the tail vein at 0, 15, 30, 45, 60, 90, and 120 minutes after glucose injection. Blood glucose levels were further detected by an Accu-Chek Performa blood glucose meter (Roche, Basel, Switzerland).

### Serum biochemical parameter analysis

Serum alanine aminotransferase (ALT), aspartate aminotransferase (AST), alkaline phosphatase (ALP), and total cholesterol (TC) were measured by a dry chemistry analyzer (FUJI DRI‐CHEM 7000 V, FUJIFILM, Tokyo, Japan) according to the manufacturer’s instructions.

### Fecal LPS concentration detection

Fecal LPS concentrations were determined by a limulus amebocyte lysate (LAL) chromogenic endpoint assay kit (Hycult Biotech, Uden, the Netherlands) according to the manufacturer’s protocols.

### Liver histology and immunohistochemistry

Portions of fresh liver were formalin-fixed and paraffin-embedded and subsequently subjected to hematoxylin and eosin (H&E) and Sirius red staining. IHC was conducted as previously described. Briefly, paraffin-embedded liver sections were deparaffinized and rehydrated, followed by incubation with anti-F4/80 antibody (Cell Signaling Technology, Danvers, MA, USA). For Oil red O staining, frozen liver portions were stained with Oil red O dye (Sigma, St. Louis, MO, USA) according to the manufacturer’s protocols. Three independent visual fields were randomly selected from each slide at 20× magnification and analyzed using ImageJ software. Pathological features were estimated according to the NAFLD activity score (NAS), Sirius red area, Oil red O staining area, and F4/80 staining intensity.

### Flow cytometry analysis

Fresh liver tissues were dissociated using a gentleMACS Dissociator (Miltenyi Biotec) and mouse Liver Dissociation Kit (Miltenyi Biotec, Bergisch Gladbach Germany). After separation and purification from the cell suspension by Percoll gradient centrifugation, liver immune cells were stained with antibodies and then subjected to a CytoFLEX LX cytometer (Beckman Coulter, CA, USA). The antibodies used in flow cytometry are listed in Table S1.

### 16S rRNA gene sequencing

Fecal DNA was extracted with a DNeasyPowerSoil Pro Kit (QIAGEN, cat. no. 47014) following the manufacturer’s protocols. Specific primers (Table S2) were applied to amplify the V3-V4 variable region of the 16S rRNA gene. The amplicon was sequenced on the Illumina NovaSeq 6000 platform. The generated raw sequencing reads were merged, denoised, and filtered to obtain amplicon sequence variants (ASVs). The obtained ASVs were assigned and annotated using the Silva database.

### Fecal untargeted metabolomics analysis

Fecal samples (30 mg) were suspended in 300 µL of methanol and fully homogenized. After centrifugation, an aliquot of 150 µL of supernatant was dried under vacuum and then incubated with methoxylamine hydrochloride (15 mg/mL in pyridine) and N,O-bis (trimethylsilyl)-trifluoroacetamide (BSTFA) containing 1% trimethylchlorosilane (TMCS) for derivatization. The derivatized extracts were subjected to gas chromatography-mass spectrometry (GC-MS) analysis using an Agilent 7890B gas chromatograph system coupled with a 5977C mass spectrometer detector (Agilent Technologies, Santa Clara, CA, USA). Metabolic data were identified and annotated by matching with the NIST 17 database and then imported into SIMCA-P software for orthogonal partial least-squares-discriminant analysis (OPLS-DA). The variable importance in projection (VIP) value was calculated in the OPLS-DA model to evaluate the variable’s contribution to the discrimination.

### Targeted quantification of tryptophan metabolites

The quantification of tryptophan and representative tryptophan metabolites in feces and serum was conducted by targeted liquid chromatography-tandem mass spectrometry (LC-MS/MS)-based metabolomics. The metabolites were extracted from 30 mg of feces or 40 μL of serum using 100 µL of methanol containing 0.1% formic acid. After filtration, samples were processed with an AB ExionLC system coupled with an AB Sciex Qtrap 6500+ mass spectrometer for LC-MS/MS analysis. Chromatographic peaks of tryptophan and tryptophan metabolites were identified based on the retention times, and metabolite concentrations were determined by comparing the peak areas of calibration curves constructed from standards.

### RNA extraction

RNA extraction of the tissues was performed with a QIAGEN RNeasy Plus Mini Kit (Qiagen, Hilden, Germany). The quality and quantity of RNA were evaluated by a NanoDrop spectrophotometer (Thermo Fisher Scientific, MA, USA). RNA integrity was assessed by an Agilent 2100 Bioanalyzer (Agilent Technologies). High-quality RNA (RNA concentration ≥ 2500 ng/μL and RIN ≥ 7) was subjected to RNA sequencing.

### Transcriptome sequencing

After being purified, enriched, and fragmented, mRNA was used to generate an mRNA library with a TruSeq Stranded mRNA LT Sample Prep Kit (Illumina, CA, USA). Sequencing was performed on the Illumina HiSeq 2500 platform (Illumina) to acquire 150-bp paired-end reads. Raw sequencing reads were filtered into clean reads using Trimmomatic and mapped to the reference genome using hisat2. The DESeq2 package in R was employed to identify the differentially expressed genes (DEGs), and these DEGs were then mapped to the Kyoto Encyclopedia of Genes and Genomes (KEGG) database for pathway enrichment analysis.

### Statistical analysis

Statistical analyses were completed using SPSS 20.0 (SPSS Inc., Chicago, IL, USA). Data are presented as the means ± standard deviations (SDs). One-way analysis of variance (ANOVA) with Fisher’s LSD post hoc test was adopted to evaluate differences among groups. A *p* value < 0.05 indicated statistical significance. Figures were plotted using GraphPad Prism 7.0 (GraphPad Software Inc., San Diego, CA, USA) and R software.

## Supplementary Material

Supplemental Material

## Data Availability

The 16S rRNA gene sequencing data are available at NCBI’s Sequence Read Archive (SRA) database under the BioProject accession code PRJNA923159 at https://www.ncbi.nlm.nih.gov/bioproject/PRJNA923159 and PRJNA951532 at https://www.ncbi.nlm.nih.gov/bioproject/PRJNA951532. The raw data of RNA sequencing are available at SRA database under the BioProject accession code PRJNA923592 at https://www.ncbi.nlm.nih.gov/bioproject/PRJNA923592.
