# Supplementary material for: Resynchronized rhythmic oscillations of gut microbiota drive time-restricted feeding induced nonalcoholic steatohepatitis alleviation
Source: Gut Microbes. 2023 Jun 12;15(1):2221450. doi: 10.1080/19490976.2023.2221450 (PMC10266122; doi:10.1080/19490976.2023.2221450)
Supplement: Supplemental Material [file KGMI_A_2221450_SM7875.pdf]

Supplementary materials

Resynchronized Rhythmic Oscillations of Gut Microbiota Drive Time-Restricted Feeding Induced Nonalcoholic Steatohepatitis Alleviation

Supplementary Table S1 List of the antibodies we used.

Antibody name	Catalog number	Manufacturer
F4/80 Rabbit mAb	70076S	Cell Signaling Technology
Goat Anti-Rabbit IgG H&L (HRP)	ab6721	Abcam
Ms CD45 APC-Cy7 30-F11	557659	BD Pharmingen
Ms CD11b FITC M1/70	557396	BD Pharmingen
Ms F4/80 PE T45-2342	565410	BD Pharmingen
Ms CD86 PE-Cy7 GL1	560582	BD Pharmingen
Ms CD16/CD32 Pure 2.4G2	553141	BD Pharmingen

Supplementary Table S2 Primer pairs of 16S rRNA sequencing.

16S rRNA universal primer	Forward primer sequence (5'-3')	Reverse primer sequence (5'-3')
Bacteria16S rRNA V3V4	TACGGRAGGCAGCAG	AGGGTATCTAATCCT

Supplementary Table S3. List of differential metabolites.

Differential metabolites in WDAL vs. NDAL	Log2FC	VIP	HMDB
(4-Hydroxy-3-Methoxyphenyl)Ethanol	-3.47414	2.55997	HMDB0038925
3-Hydroxypicolinic Acid	-4.75362	2.36269	HMDB0013188
3-Hydroxyphenylacetic Acid	-4.22195	2.30722	HMDB0000440
Cholesterol	2.955787	2.29783	HMDB0000067
Resveratrol	-3.99053	2.2821	HMDB0003747
5-Hydroxy-3-Indoleacetic Acid	-4.16714	2.25753	HMDB0000763
5-Hydroxyindoleacetic Acid	-4.16052	2.25228	HMDB0000763
D-Ribose	-3.99207	2.19851	HMDB0000283
Pinitol	-4.16059	2.14545	HMDB0034219
D-Xylose	-4.13816	2.14369	HMDB0000098
1-Monolinolein	-4.09815	2.13198	HMDB0011568
2-(4-Methyl-1-Piperazinyl)Ethanamine	-3.73724	2.08583	HMDB0062673
4-Hydroxyproline	-2.34364	2.08411	HMDB0000725
Myristic Acid	3.909466	2.06814	HMDB0000806
2-Hydroxy-2-Phenylpropanoic Acid	-4.56982	2.05465	HMDB0142137
Galacturonic Acid	-3.72189	2.04436	HMDB0002545
D-Arabinose	-3.73281	2.03708	HMDB0029942
Dodecanoic Acid	3.973602	2.02418	HMDB0000638
2',6'-Dihydroxyacetophenone	-4.21553	2.00768	HMDB0029660
Kynurenic Acid	-3.46178	1.9972	HMDB0000715
3,4-Dihydroxycinnamic Acid	-3.25142	1.99313	HMDB0001964
Delta-Tocopherol	-3.46689	1.98433	HMDB0002902
Capric Acid	3.752766	1.97148	HMDB0000511
2-Monoolein	-3.46041	1.96719	HMDB0011537
Enterolactone	-3.23586	1.96266	HMDB0006101
Maltotriose	-4.08241	1.93871	HMDB0001262
Methyl Beta-D-Glucopyranoside	-4.49474	1.91247	HMDB0029965
Trehalose	-3.83676	1.9109	HMDB0000975
Isoferulic Acid	-3.21064	1.86643	HMDB0000955
3-(3-Hydroxyphenyl)Propionic Acid	-3.51191	1.85194	HMDB0000375
Scopoletin	-3.1743	1.83531	HMDB0034344
4-Aminophenol	-3.53784	1.82684	HMDB0001169
7-Ketocholesterol	3.030157	1.811	HMDB0000501
Lathosterol	2.89671	1.80185	HMDB0001170
L-Lactic Acid	-2.93052	1.79698	HMDB0000190

2-Methylglyceric Acid	-3.01238	1.77672	HMDB0002601
3-Hydroxypalmitic Acid	-2.42024	1.77281	HMDB0010734
Bisphenol A	3.873272	1.76231	HMDB0032133
Stigmastanol	-2.70673	1.76009	HMDB0000494
Leucinic Acid	-3.48501	1.73964	HMDB0000665
Threose	3.001224	1.72458	HMDB0002649
Tocopherol Acetate	-2.88443	1.72178	HMDB0033685
Monooleoylglycerol	-2.9234	1.71654	HMDB0011567
2-Hydroxy-3-Methylbutyric Acid	-3.6923	1.65224	HMDB0000407
2-Hydroxycaproic Acid	-3.81681	1.64271	HMDB0001624
N-Acetyl-D-Mannosamine	-2.18007	1.585	HMDB0001129
Linoleic Acid	-2.25736	1.58211	HMDB0000673
N-Acetyl-5-Hydroxytryptamine	-3.68172	1.53768	HMDB0001238
Sebacic Acid	2.450067	1.51283	HMDB0000792
Galactinol	-2.43107	1.46271	HMDB0005826
3,5-Dihydroxybenzoic Acid	-3.50503	1.45396	HMDB0013677
Dihydroxyacetone	-2.31274	1.41237	HMDB0001882
Tridecanoic Acid	2.029597	1.40403	HMDB0000910
Daidzein	-2.16819	1.39543	HMDB0003312
Benzenepropanoic Acid	-1.71614	1.39403	HMDB0000158
Cadaverine	-1.95828	1.39222	HMDB0002322
Alpha-Ketoglutarate	-1.93553	1.37966	HMDB0000208
3-Hydroxymethylglutaric Acid	-1.83049	1.37047	HMDB0000355
Quinic Acid	-2.35965	1.36054	HMDB0003072
Behenic Acid	-1.78709	1.33989	HMDB0000944
Glutaric Acid	-1.97165	1.33294	HMDB0000661
Aspartate	2.54255	1.3292	HMDB0000191
Syringic Acid	-2.58398	1.31433	HMDB0002085
Hypoxanthine	1.426562	1.30441	HMDB0000157
Menthol	-1.53601	1.29483	HMDB0003352
11-Eicosenoic Acid	1.852992	1.28318	HMDB0034296
Butane-2,3-Diol	-2.33948	1.26095	HMDB0003156
N-Butyrylglycine	-1.84019	1.26095	HMDB0000808
Beta-Sitosterol	-1.6266	1.26007	HMDB0000852
Xylonolactone	-1.7924	1.25966	HMDB0011676
Noradrenaline	-1.55359	1.24754	HMDB0000216
5beta-Coprostanol	1.70534	1.24286	HMDB0000577
Phenol	-1.6401	1.2322	HMDB0000228
Docosahexaenoic Acid	-1.57634	1.22638	HMDB0002183
D-Xylitol	-1.66374	1.20809	HMDB0002917
D-Ribose 5-Phosphate	-1.70533	1.20757	HMDB0001548
1-Methylhydantoin	-1.93229	1.19925	HMDB0003646
Indoleacetic Acid	-1.51227	1.18529	HMDB0000197
2-Hydroxybutyric Acid	-1.98857	1.1846	HMDB0000008
Sedoheptulose	-2.29678	1.17947	HMDB0003219
Tetracosanoic Acid	-1.43845	1.1782	HMDB0002003
Cholic Acid	1.862801	1.17776	HMDB0000619

L-Threonine	1.518989	1.15484	HMDB0000167
Lignoceric Acid	-1.8286	1.13125	HMDB0002003
Malic Acid	-1.61375	1.12092	HMDB0000156
Cis-Gondoic Acid	1.339164	1.11259	HMDB0002231
Hexaric Acid	-1.41131	1.11166	HMDB0000639
D-Fructose	-1.27943	1.10464	HMDB0000660
O-Phosphoserine	1.621558	1.10208	HMDB0000272
Batyl Alcohol	1.986623	1.09987	HMDB0011143
3-(4-Hydroxy-3-Methoxyphenyl)Propionic Acid	-1.44299	1.09264	HMDB0062121
L-2-Hydroxyglutaric Acid	-1.94606	1.07892	HMDB0000694
Lactitol	-1.62275	1.07009	HMDB0040937
Phytanic Acid	2.314252	1.06827	HMDB0000801
Palmitelaidic Acid	1.21824	1.06755	HMDB0012328
Beta-Hydroxymyristic Acid	1.775534	1.06746	HMDB0010731
25-Hydroxycholesterol	1.190473	1.05861	HMDB0006247
Urea	-1.60263	1.05672	HMDB0000294
Citramalic Acid	-1.83276	1.05522	HMDB0000426
L-Isoleucine	3.314515	1.05256	HMDB0000172
3,4-Dihydroxyphenylglycol	1.54816	1.04854	HMDB0000318
Cellobiose	-1.18486	1.03694	HMDB0000055
O-Phosphoethanolamine	1.35654	1.03643	HMDB0000224
Coniferin	-2.16045	1.03199	HMDB0013682
2-Palmitoylglycerol	-1.31128	1.02362	HMDB0011533
Pentadecanol	2.946885	1.02263	HMDB0013299
Palmitoleic Acid	1.160026	1.01872	HMDB0003229
Sorbitol	-2.30679	1.0139	HMDB0000247
Pyrazin-2-Carboxylic Acid	-1.97182	1.01366	HMDB0059734
2-Pyrrolidinone	-1.08339	1.01282	HMDB0002039
L-Aspartic Acid	1.761648	1.01093	HMDB0000191
1-Octacosanol	1.211897	1.00308	HMDB0034380
Galactitol	-0.99056	1.00111	HMDB0000107
Alpha-Lactose	-2.26953	1.0008	HMDB0000186

Differential metabolites in WDTRF vs. WDAL	Log2FC	VIP	HMDB
1-Methylhydantoin	2.412462	2.51536	HMDB0003646
Hydroxypropionic Acid	-0.97894	1.58346	HMDB0000700
2-Hydroxypentanoic Acid	-0.66627	1.3334	HMDB0001863
3-Methyl-2-Oxovaleric Acid	0.483672	1.3814	HMDB0000491
3-Aminoisobutyric Acid	-0.79446	2.38849	HMDB0003911
L-Isoleucine	-1.41522	1.75081	HMDB0000172
Trans-4-Hydroxyproline	-0.88152	1.96334	HMDB0000725
L-Proline	-1.71575	3.13206	HMDB0000162
Glycine	0.033713	1.02586	HMDB0000123
4-Nitrophenol	-1.16465	1.71042	HMDB0001232
5-Hydroxypentanoic Acid	-0.85752	1.16899	HMDB0061927
Serine	-0.42286	1.49408	HMDB0000187
Hydrocinnamic Acid	-1.13248	2.24277	HMDB0000764
Benzenepropanoic Acid	1.640421	2.79771	HMDB0000158
Aspartate	-1.03243	1.17048	HMDB0000191
3-Amino-2-Piperidone	-1.20745	1.96895	HMDB0000323
D-Erythro-Sphingosine	-1.07752	1.95006	HMDB0000252
L-Methionine	-2.01574	3.13565	HMDB0000696
L-Aspartic Acid	-1.1029	1.45036	HMDB0000191
Oxoproline	-0.45498	1.02144	HMDB0000267
2-Hydroxyadipic Acid	0.853795	1.40418	HMDB0000321
Phenylpyruvic Acid	0.368101	1.56528	HMDB0000205
O-Phosphoserine	-0.46413	1.32834	HMDB0000272
N-Acetylaspartate	-0.60921	1.05583	HMDB0000812
Cis-4-Hydroxycyclohexylacetic Acid	-3.28621	5.5407	HMDB0000451
L-Glutamic Acid	-0.45262	1.09859	HMDB0000148
Dl-Dopa	-0.90149	1.45759	HMDB0000609
Galactitol	-0.78621	1.04887	HMDB0000107
D-Xylose	0.994571	1.28511	HMDB0000098
D-Xylulose	2.35576	3.44365	HMDB0001644
Digalacturonic Acid	-1.68396	1.28738	HMDB0039721
3-(3-Hydroxyphenyl)Propionic Acid	0.95759	1.69796	HMDB0000375
4-Aminophenol	0.998391	1.68252	HMDB0001169
Phosphoethanolamine	-0.74975	1.01326	HMDB0000224
O-Phosphoethanolamine	-0.87016	1.09379	HMDB0000224
Serotonin	-0.53223	1.01433	HMDB0000259
Hypoxanthine	-4.431	4.64115	HMDB0000157
Ornithine	-1.01647	1.38565	HMDB0000214
Noradrenaline	1.302351	2.30407	HMDB0000216
Pinitol	5.531581	5.13865	HMDB0034219
Pentadecanol	0.491353	1.22637	HMDB0013299
Leucyl-Glycine	-0.85874	1.50554	HMDB0028929

D-Fucose	-0.79242	1.4671	HMDB0029196
L-Lysine	-1.4438	2.49713	HMDB0000182
Norvaline	-0.58626	1.33015	HMDB0013716
L-Tyrosine	-0.28817	1.16179	HMDB0000158
Glucosamine	-0.48759	1.03962	HMDB0001514
Pentacosanoic Acid	-1.58678	2.67424	HMDB0002361
Galactose	-0.67597	1.3295	HMDB0000143
Maltitol	-0.76548	1.10529	HMDB0002928
Shikimic Acid	-0.82279	1.20229	HMDB0003070
Palmitic Acid	-0.33703	1.21337	HMDB0000220
Isoferulic Acid	0.802607	1.30698	HMDB0000955
Heptadecanoic Acid	-1.39644	2.43209	HMDB0002259
Cerotinic Acid	-0.57705	1.48657	HMDB0002356
Lignoceric Acid	1.561973	1.27448	HMDB0002003
Phytol	-0.69448	1.33747	HMDB0002019
Spermine	-0.51904	1.01394	HMDB0001256
Phytanic Acid	-1.28411	1.16825	HMDB0000801
L-Tryptophan	-0.62239	1.12182	HMDB0000929
D-Fructose-1-Phosphate	0.615609	1.27799	HMDB0000399
Biphenyl	0.480943	1.05938	HMDB0034437
Arachidyl Alcohol	0.367922	1.17364	HMDB0011619
Saccharopine	1.022527	1.70368	HMDB0000279
Menthol	0.435367	1.04748	HMDB0003352
Inosine	-0.0016	1.26349	HMDB0000195
1-Monopalmitin	-0.55931	1.45806	HMDB0011564
(4-Hydroxy-3-Methoxyphenyl)Ethanol	3.614643	5.53491	HMDB0038925
Batyl Alcohol	-1.21728	1.21723	HMDB0011143
1-Monostearin	0.488551	1.05543	HMDB0011131
5'-Deoxy-5'-Methylthioadenosine	-0.80572	1.74977	HMDB0001173
Daidzein	1.173609	1.98382	HMDB0003312
5alpha-Cholestanol	0.902544	2.59141	HMDB0000908
Norethindrone	0.956896	2.33863	HMDB0014855
25-Hydroxycholesterol	-0.59193	1.26513	HMDB0006247
Lanosterol	-2.25621	3.43436	HMDB0001251

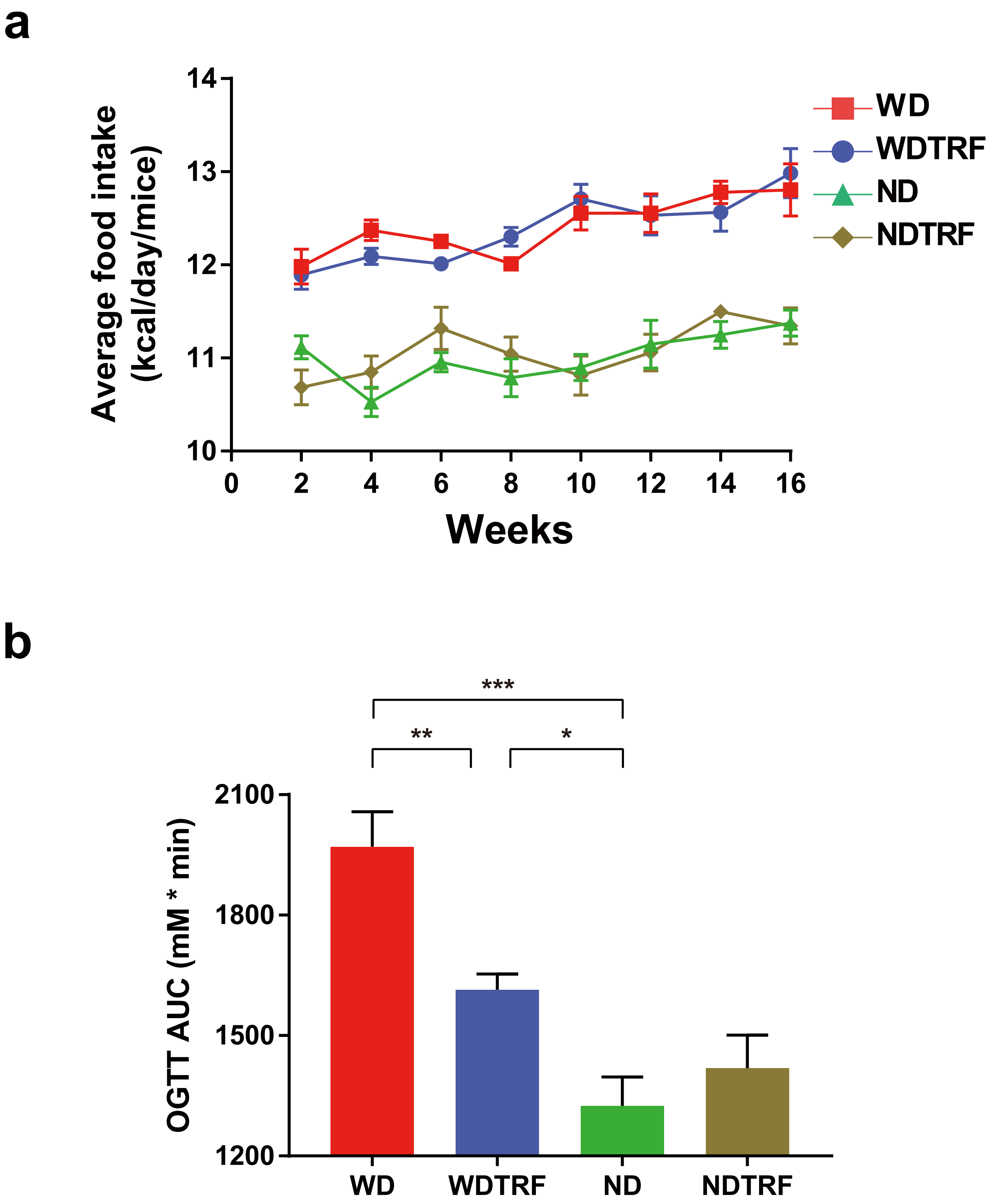

Supplementary Figure S1. (a) Average food intake curve. (b) Area under OGTT curve. n = 10 in each group. The data are presented as the mean ± SEM. *$p < 0.05$; **$p < 0.01$, ***$p < 0.001$ for the comparison.

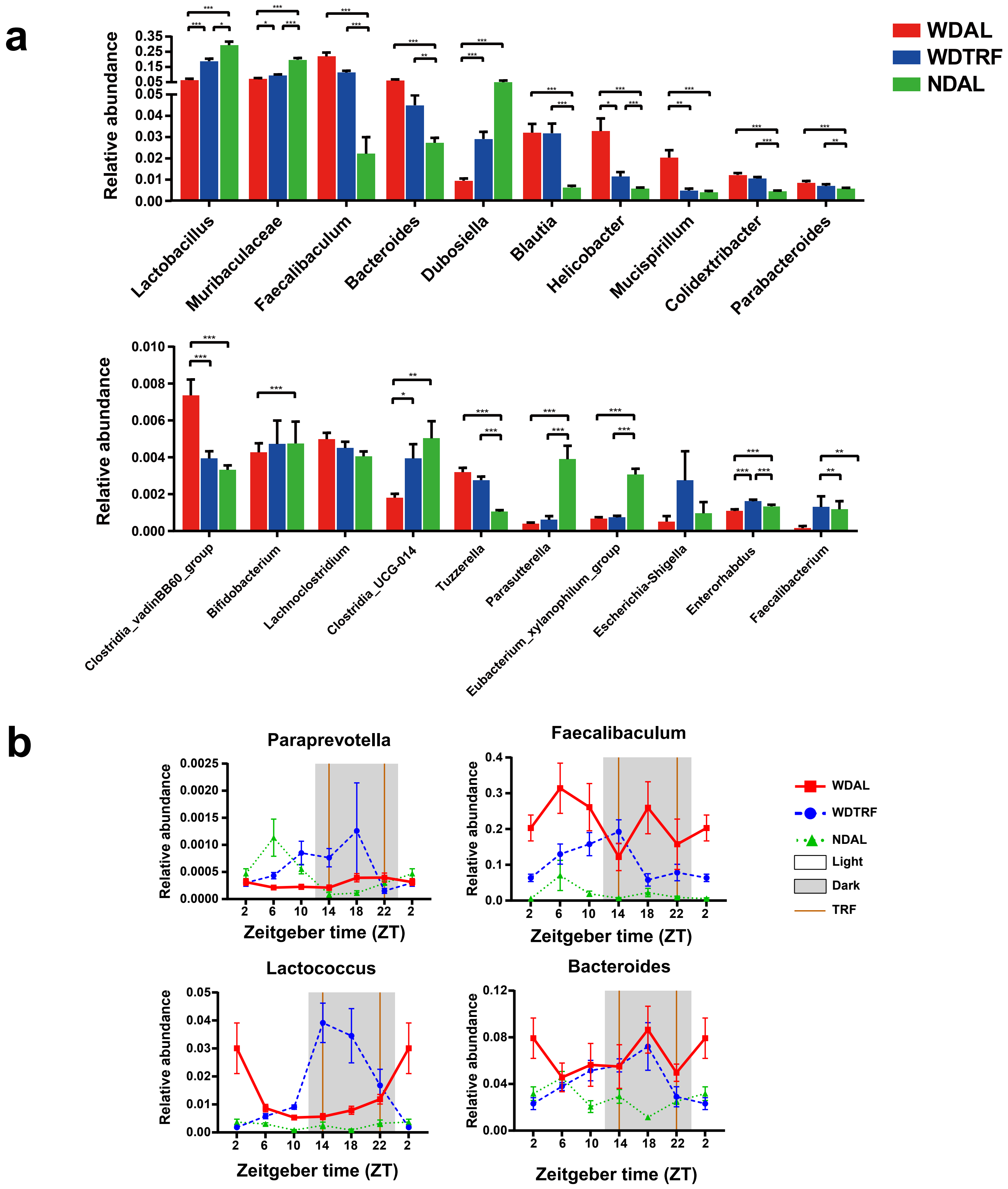

Supplementary Figure S2. (a) Relative abundance of significantly changed genera (Kruskal-Wallis test). n = 48 in each group. (b) Diurnal oscillations of several genera over the course of a day. n = 8 at each time point in each group. The data are presented as the mean ± SEM. *p < 0.05; **p < 0.01, ***p < 0.001 for the comparison.

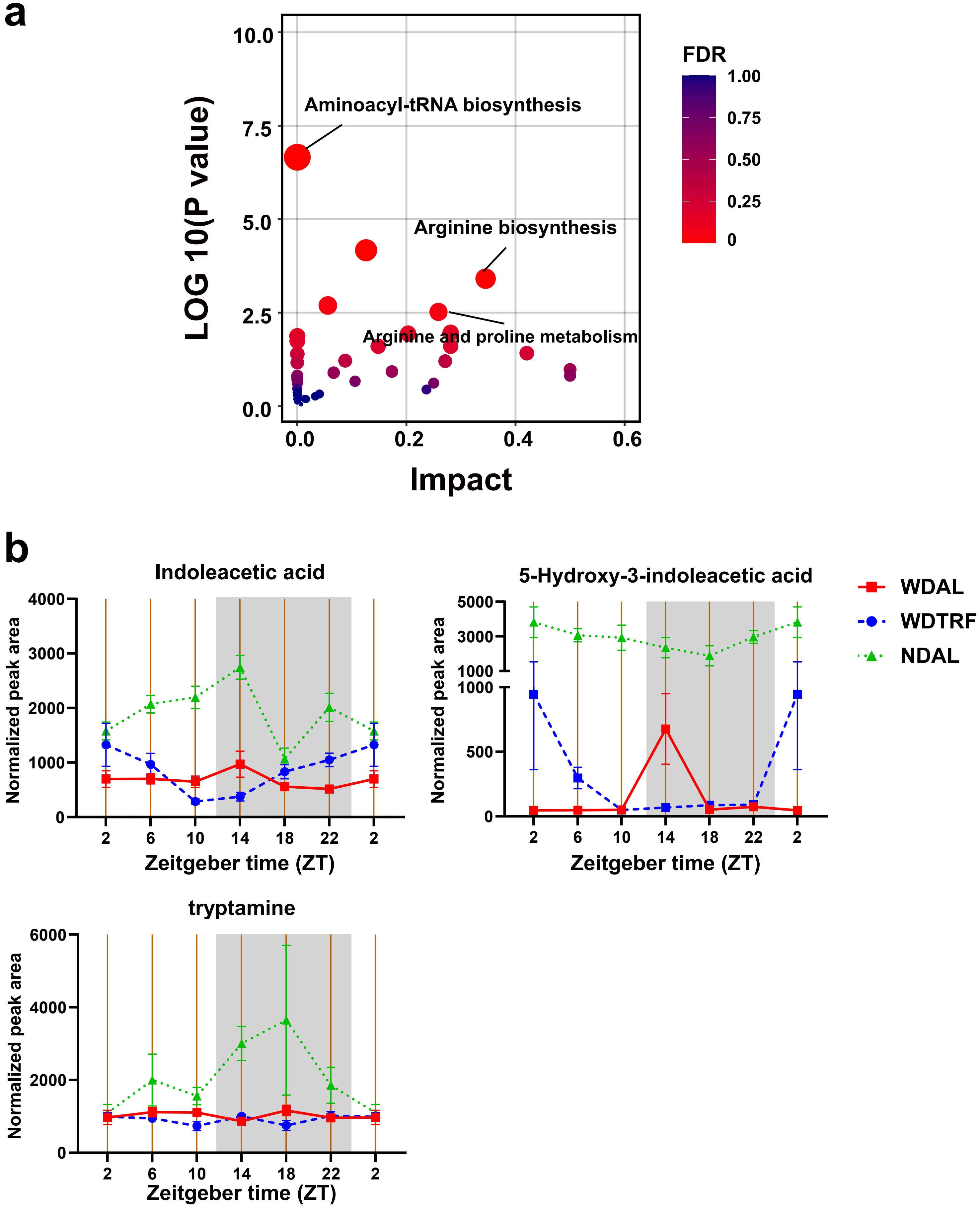

Supplementary Figure S3. (a) KEGG pathway enrichment analysis of rhythmic metabolites in NDAL group. (b) Diurnal oscillations of indole derivatives. n = 6 at each time point in each group.

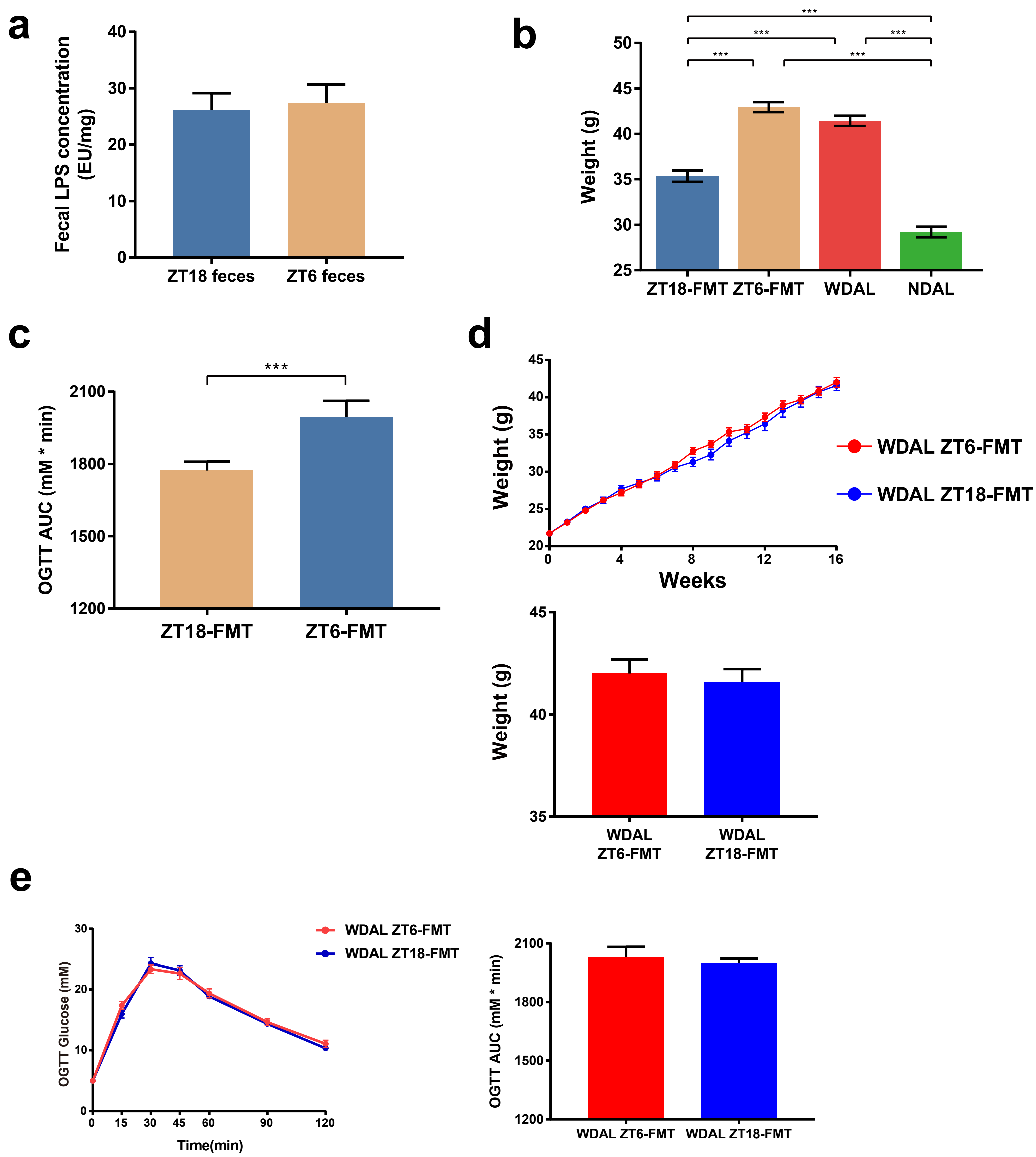

Supplementary Figure S4. (a) WDTRF fecal LPS concentrations at ZT18 and ZT6. (b) Body weight after 16 weeks feeding. (c) Area under OGTT curve of ZT18-FMT and ZT6-FMT. (d) Body weight curve of WDAL ZT6-FMT and WDAL ZT18-FMT (upper panel) and body weight after 16 weeks feeding (lower panel). (e) OGTT curve of WDAL ZT6-FMT and WDAL ZT18-FMT (left panel) and area under OGTT curve (right panel). n = 8 in each group. The data are presented as the mean ± SEM. *p < 0.05; **p < 0.01, ***p < 0.001 for the comparison.

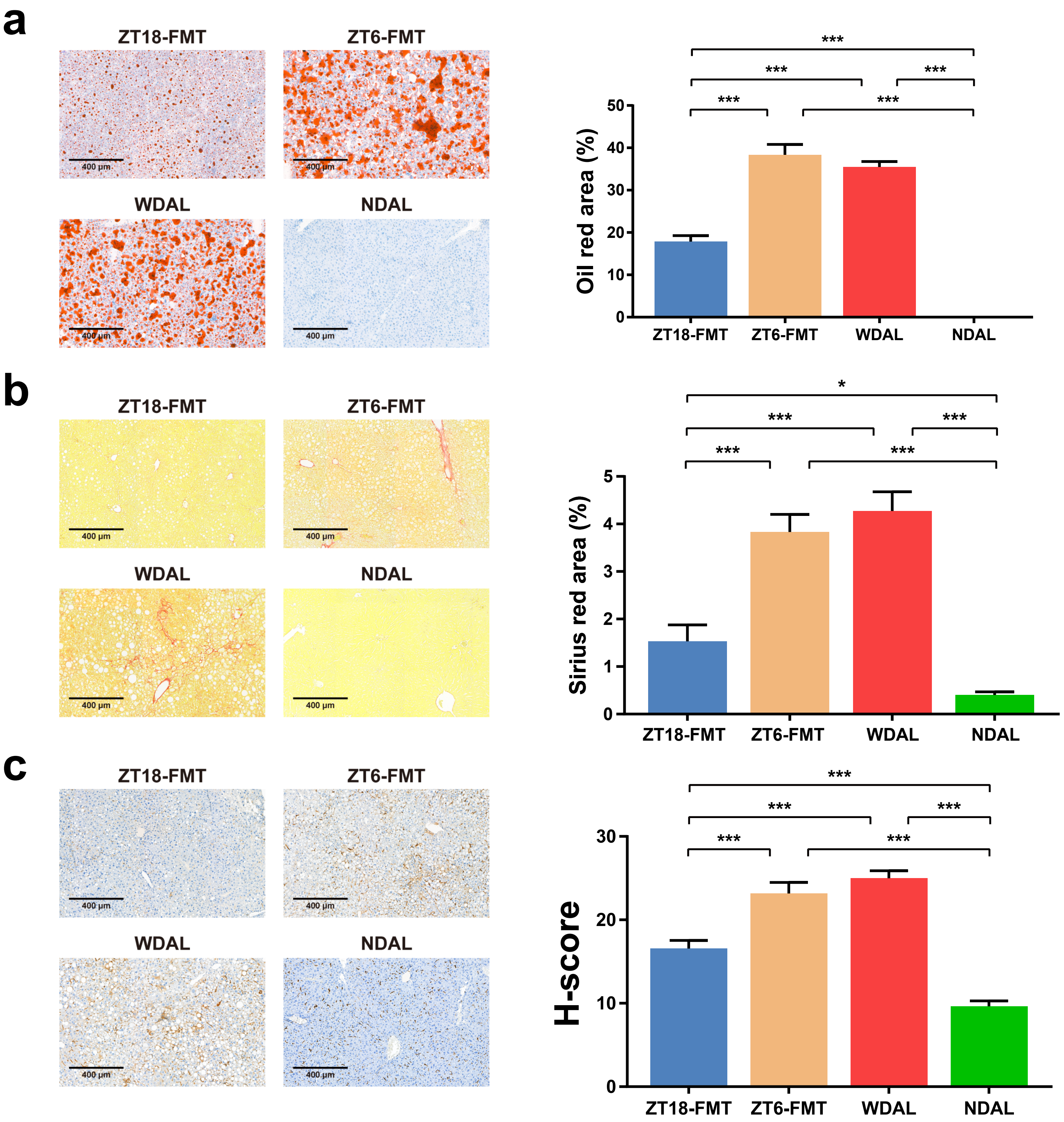

Supplementary Figure S5. (a) Representative images of Oil Red O staining of liver (upper panel, original magnification × 20, scale bar = 400 mm) and quantitative analysis of Oil red O area (lower panel). (b) Representative images of Sirius red staining of liver (upper panel, original magnification × 20, scale bar = 400 mm) and quantitative analysis of Sirius red area (lower panel). (c) Representative images of immunohistochemical staining of F4/80 (upper panel, original magnification × 20, scale bar = 400 mm) and H-score (lower panel). The H-score was assessed by ImageJ, demonstrating the staining intensity. n = 8 in each group. The data are presented as the mean ± SEM. $^{*}p < 0.05$; $^{**}p < 0.01$, $^{***}p < 0.001$ for the comparison.

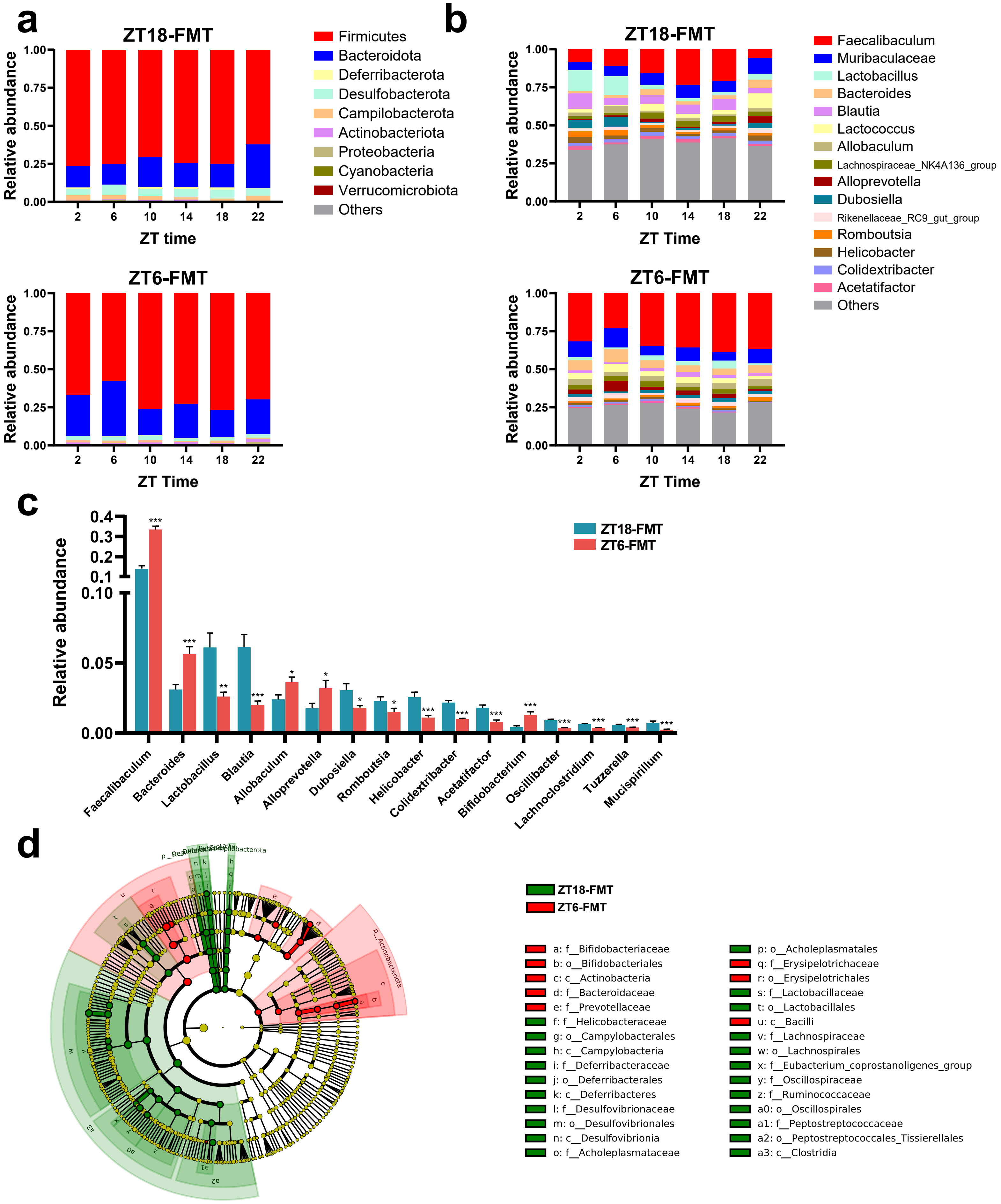

Supplementary Figure S6. (a) The top 9 most abundant phyla in ZT6-FMT and ZT18-FMT. (b) The top 15 most abundant genera in ZT6-FMT and ZT18-FMT. (c) Relative abundances of significantly changed genera (Mann-Whitney test). (d) LEfSe cladogram with LDA score > 3.5 in LEfSe analysis. n = 6 at each time point in each group.The data are presented as the mean ± SEM. $*p < 0.05$; $**p < 0.01$, $***p < 0.001$ for the comparison.

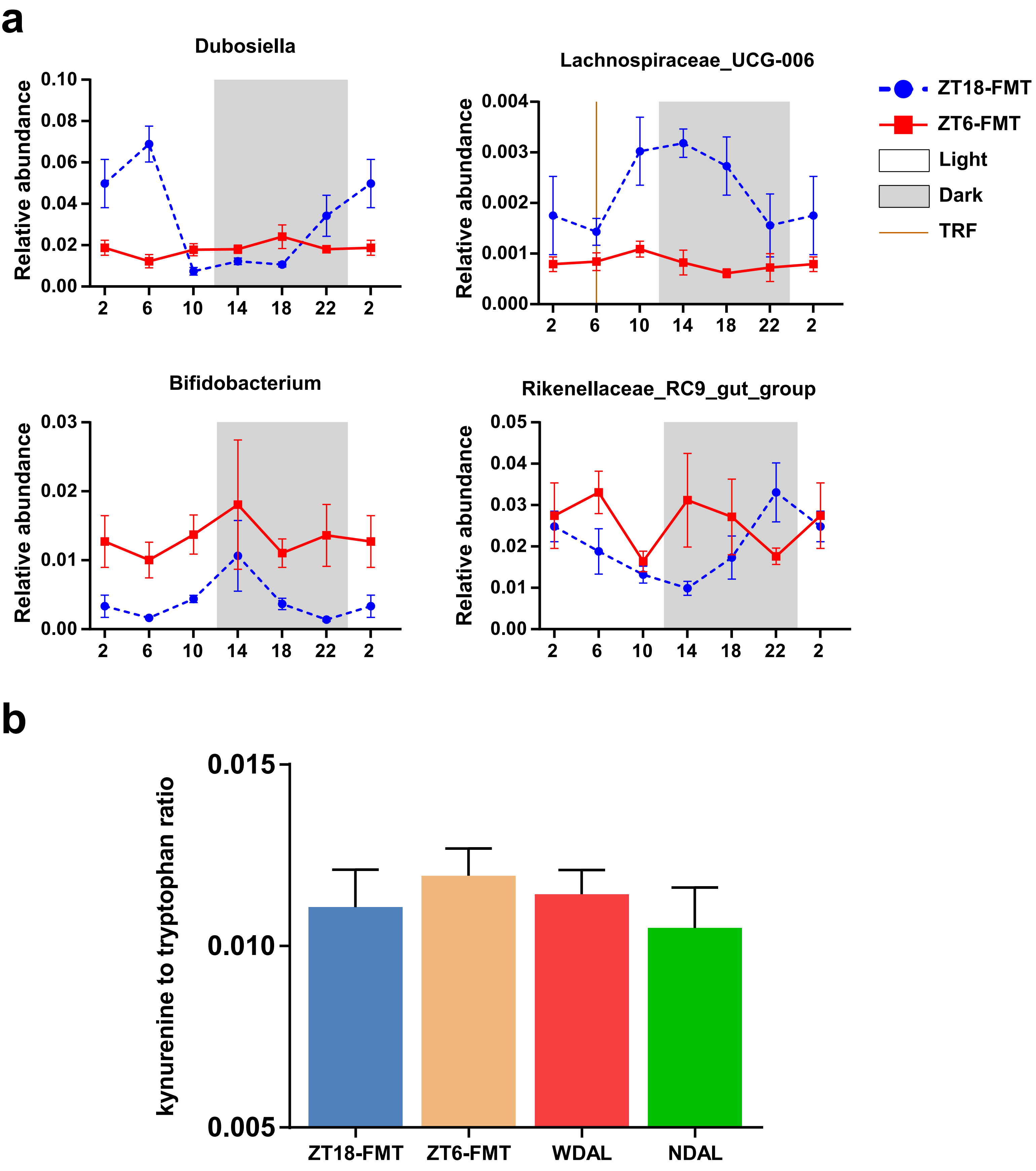

Supplementary Figure S7. (a) Diurnal oscillations of several genera over the course of a day. n = 6 at each time point in each group. (b) Kynurenine to tryptophan ratio. n = 8 in each group. The data are presented as the mean ± SEM.
